# Ring-opening copolymerization of 2-vinyloxirane with anhydride/carbon dioxide: facile access to backbone-editable polymers with tunable lifecycles

**DOI:** 10.1093/nsr/nwaf534

**Published:** 2025-11-28

**Authors:** Mingxin Niu, Chenyang Hu, Zhenbiao Xie, Qi Zhang, Bokun Li, Zhiqiang Sun, Xuan Pang, Xuesi Chen

**Affiliations:** State Key Laboratory of Polymer Science and Technology, Changchun Institute of Applied Chemistry, Chinese Academy of Sciences, Changchun 130022, China; School of Applied Chemistry and Engineering, University of Science and Technology of China, Hefei 230026, China; State Key Laboratory of Polymer Science and Technology, Changchun Institute of Applied Chemistry, Chinese Academy of Sciences, Changchun 130022, China; State Key Laboratory of Polymer Science and Technology, Changchun Institute of Applied Chemistry, Chinese Academy of Sciences, Changchun 130022, China; School of Applied Chemistry and Engineering, University of Science and Technology of China, Hefei 230026, China; State Key Laboratory of Polymer Science and Technology, Changchun Institute of Applied Chemistry, Chinese Academy of Sciences, Changchun 130022, China; School of Applied Chemistry and Engineering, University of Science and Technology of China, Hefei 230026, China; State Key Laboratory of Polymer Science and Technology, Changchun Institute of Applied Chemistry, Chinese Academy of Sciences, Changchun 130022, China; School of Applied Chemistry and Engineering, University of Science and Technology of China, Hefei 230026, China; State Key Laboratory of Polymer Science and Technology, Changchun Institute of Applied Chemistry, Chinese Academy of Sciences, Changchun 130022, China; State Key Laboratory of Polymer Science and Technology, Changchun Institute of Applied Chemistry, Chinese Academy of Sciences, Changchun 130022, China; School of Applied Chemistry and Engineering, University of Science and Technology of China, Hefei 230026, China; State Key Laboratory of Polymer Science and Technology, Changchun Institute of Applied Chemistry, Chinese Academy of Sciences, Changchun 130022, China; School of Applied Chemistry and Engineering, University of Science and Technology of China, Hefei 230026, China

**Keywords:** polyesters, ring-opening copolymerization, polymer backbone modification, lifecycle modulation

## Abstract

Polymer backbone modification (PBM) is an emerging strategy uniquely suited to tuning the intrinsic properties of polymers. However, its utilization in tuning the degradability of polymers is underexplored, and there is no viable route to backbone-editable polyesters and polycarbonates with tunable lifecycles. In this work, we synthesized a series of backbone-editable polyesters and polycarbonate via ring-opening copolymerization (ROCOP) of 2-vinyloxirane (VIO) with anhydrides/CO_2_. These polymers feature the specific structure necessary for [3,3]-sigmatropic oxo-rearrangements under a Pd catalyst, in which the terminal olefins can undergo rearrangement to *trans* internal ones and facilitate backbone modification. After optimizing the rearrangement conditions, we were able to rearrange the polyesters in satisfactory yields (55.2%–72.3%) without affecting the molecular weight. Notably, compared with the original polymers, the rearranged ones exhibit lower hydroboration–oxidation reactivity and glass transition temperature, as well as much faster thermal and hydrolytic degradation profiles, providing a new strategy for the design of polymers with tunable properties and lifecycles.

## Teaser

This study synthesized a series of backbone-editable polymers via ring-opening copolymerization, which can successfully regulate the performance and life cycle of the synthesized polymers with inherent molecular weight.

## INTRODUCTION

Chemical modification is a key process in modulating the properties and functionalities of polymers. While this process has been centered overwhelmingly on the polymer periphery, attempts to edit the backbone of polymers are quite limited [1]. As the core component, the backbone plays a fundamental role in a polymer, and polymer backbone modification (PBM) would induce dramatic changes of material properties including degradability. Meanwhile, the editing of skeletal composition also opens avenues to a wide variety of polymer structures that are not accessible through traditional synthetic routes. The early traceable demonstrations of PBM were published in the 1960s, in which nitrogen atoms were inserted into polyketones through Schmidt or Beckmann rearrangements [2,3]. In 2004, Kosaka *et al.* pioneered the insertion of oxygen atoms into polyketones through Baeyer–Villiger oxidation [4,5]. Since then, PBM has emerged as an attractive tool that has gathered a surge of interest, focusing primarily on non-biodegradable polymers. For example, Chen and co-workers reported two-step catalytic oxidations of the C–H bonds in polyethylenes to incorporate ketone and alcohol units [6]. Baur *et al.* further conducted Baeyer–Villiger oxidation with high-density polyethylene bearing in-chain keto groups to insert oxygen atoms into chains [7]. Recently, by revisiting Beckmann rearrangement, Lu *et al.* synthesized long-chain polyamides from polyethyleneketones [8]. Furthermore, Galan and Brantley transformed olefin-containing polymers into polyallenes utilizing Skattebøl rearrangement [9]. Zhukhovitskiy also introduced acyl silane functionality to poly(acyl silane)s through anionic Brook rearrangements [10]. In addition, PBM utilizing force-induced ring opening of mechanophores has also received considerable attention [11, 12].

**Figure 1.**
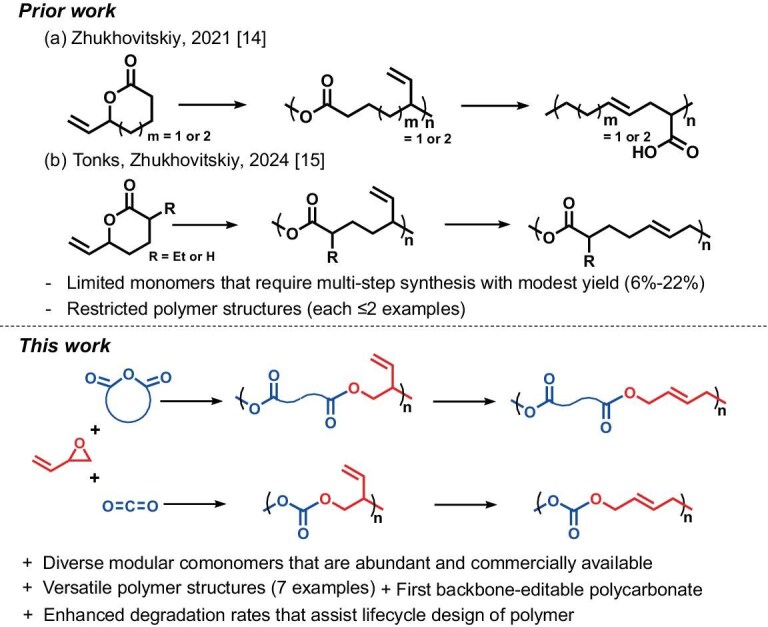
Overview of the design and preparation of backbone-editable polyesters and polycarbonates.

Aliphatic polyesters and polycarbonates are prominent categories of sustainable polymers with wide commodity and medical applications [13]. For these polymers, the development of PBM can result in unique classes of sustainable materials with tunable properties and interesting applications. However, a few attempts to harness PBM on polyesters can be found in the literature, and to the best of our knowledge there is no practical route to backbone-editable polycarbonates. In 2021, Ratushnyy and Zhukhovitskiy reported the trailblazer work of incorporating an allylic carboxylate moiety to enable the backbone of polyesters to be editable, in which the polyesters can be transformed into vinyl polymers via Ireland–Claisen sigmatropic rearrangement (Fig. 1) [14]. Subsequently, Ditzler and co-workers demonstrated that similar polyesters could also be edited via [3,3]-sigmatropic oxo-rearrangements to turn branched polyesters into linear counterparts [15]. Although elegant, these works relied on ring-opening polymerization (ROP) of ω-vinyl lactones to synthesize polyesters featuring an allylic carboxylate moiety, in which the monomers’ availability is limited by tedious synthesis and modest yield (Fig. S1). To this end, the structural diversity of backbone-editable polyesters is very restricted, precluding further structural engineering and applications. By contrast, alternating ring-opening copolymerization (ROCOP) of epoxides and anhydrides/CO_2_ to synthesize polyesters/polycarbonates is a promising approach that utilizes a broad range of commercially available comonomers, enabling access to a wider variety of sustainable polymers. However, a synthetic pathway of ROCOP to backbone-editable polyesters and polycarbonates has not been established. A key challenge is the judicious selection of the monomer sets to embed backbone-editable moieties.

Nowadays, under the ever-growing requirement for polymers with on-demand durability and lifecycles, the modulation of polymer degradation has attracted considerable scientific attention [16]. Conventionally, traditional polymeric materials including polyolefins [17], polyurethanes [18] and polyethylene terephthalate (PET) [19] show relatively low degradability, and typically can only be mechanically recycled or chemically upcycled [19,20]. By contrast, new classes of polymers with tunable degradation cycles could meet the requirements of sustainability and potentially serve broader applications. In 2023, Vidal *et al.* synthesized a series of generally applicable borate-polyesters via ROCOP. They could modulate the water degradation capacity of these polyesters by transforming the boronic acid substituents into borates. These strategies of modulating degradation capacity mostly rely on side-chain functionalization. However, the PBM strategies in ROCOP lack similar precedents [21]. As demonstrated in previous studies, the PBM method has proved to be effective in enhancing the degradation capacity of polyolefins [7,8,22]. On the other hand, we speculated that this approach also holds significant potential for modulating the degradation performance of sustainable polymers. Considering that modifying a minority of the polyester backbone can enhance its degradation efficiency [e.g. remarkable change in the degradability of poly(butylene adipate-*co*-terephthalate) (PBAT) with a small amount of oxalic acid] [23], we rationalized PBM to be a potentially efficient approach of modulating the degradability of polyesters. Further, it is believed that a proof-of-concept study on PBM toward polyesters and polycarbonates would open new avenues for designing the lifecycle of sustainable polymers.

In this work, by choosing 2-vinyloxirane (VIO) as the monomer to copolymerize with six kinds of cyclic anhydrides, as well as CO_2_ via ROCOP, we prepared a series of polyesters and polycarbonates that can undergo rearrangement reaction via [Pd] catalysis to be transformed from a branched structure to a linear structure. By further running the rearrangement reaction under diverse conditions, we optimized the PBM conversion of the obtained polymers. With the rearranged polymers in hand, we also explored the influence of rearrangement with focus on reactivity, thermal properties and degradation profiles. This research would provide valuable insights into further polymer modification and the design of polymer lifecycles.

## RESULTS AND DISCUSSION

At the outset, we synthesized a series of polyesters/polycarbonates via ROCOP of cyclic anhydrides/VIO and CO_2_/VIO. We employed [1,2-cyclohexanediamino-*N, N′*-bis(3,5-di-*t*-butylsalicylidene)]chromium(III) chloride [(salen)CrCl]/bis(triphenyl phosphine)iminium chloride (PPNCl) [24] or triethylborane (TEB)/PPNCl as the catalyst system of ROCOP reaction, because of their good activity, substrate scope and selectivity [24–29]. ROCOP of phthalic anhydride (PA) and VIO catalyzed by (salen)CrCl/PPNCl with the loadings of [(salen)CrCl]:[PPNCl]:[PA]:[VIO] = 1:1:400:600 could achieve complete PA conversion within 8 h at 80°C, leading to a molecular weight of 9.8 kDa and *Đ* of 1.34 (Table S1, entry 1). Based on the polymerization conditions, others commercially available cyclic anhydrides, including glutaric anhydride (GA), 4-bromophthalic anhydride (BPA), succinic anhydride (SA), maleic anhydride (MA) and 3-methylglutaric anhydride (mGA), were polymerized with VIO (Table S1, entries 2–6). Considering that some anhydrides may undergo side-reactions at elevated temperatures, the reaction temperature was appropriately decreased form 80°C to 60°C for GA, SA, mGA and MA. These obtained polyesters all exhibited a wide distribution (*Đ* > 1.4). While TEB/PPNCl as the catalyst and benzyl alcohol (BnOH) as the initiator were used for polymerization, ROCOP of PA/VIO would achieve complete PA conversion within 8 h and a lower dispersity (*Đ* = 1.15) for polyester **P1** (Table S1, entry 7). However, the polyester **P2** synthesized by ROCOP of GA/VIO under the same loading still exhibited a wide distribution (*Đ* = 1.51) (Table S1, entry 8). Matrix-assisted laser desorption ionization time-of-flight mass spectrometry (MALDI-TOF MS) of polyester **P2** showed that Δ*m*/*z* between two adjacent peaks was 184.2, which is the same as theoretical values (114.1 for GA and 70.1 for VIO) (Fig. S2). In addition, end-group analysis showed that the peak series could be assigned to Cl/H or PhCH_2_O/H, indicating that there are two initiation systems in ROCOP. Even when the supplementary initiator BnOH was introduced, a partial Cl^−^ (PPNCl) initiation mechanism persisted. This result could also be reflected as a bimodal distribution in gel permeation chromatography (GPC) traces of **P2**, and the end-group PhCH_2_O/H could be observed in the value of proton nuclear magnetic resonance (^1^H NMR) (Fig. S3). Besides this, we also attempted to use CO_2_ as a comonomer; the polymerization could proceed to a VIO conversion of 43.9% within 4 days (Table S1, entry 9). All obtained polyesters/polycarbonates were characterized by ^1^H NMR (Figs S3–S9), indicating that all the polymers have well-defined alternating structures containing <10% ether linkages (signals at 3.4–3.8 ppm).

**Figure 2.**
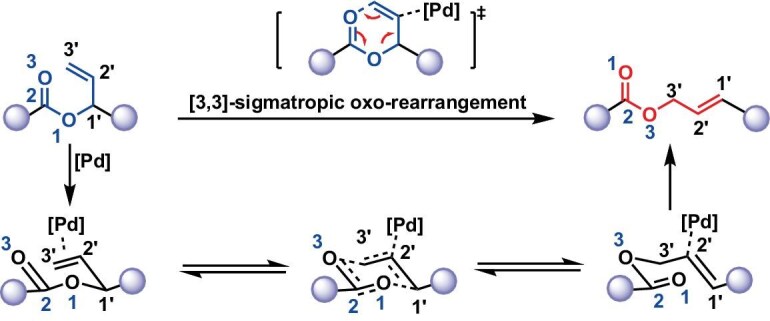
Proposed [3,3]-sigmatropic oxo-rearrangement of polyesters or polycarbonates catalyzed by [Pd] via an acetoxonium intermediate.

**Figure 3.**
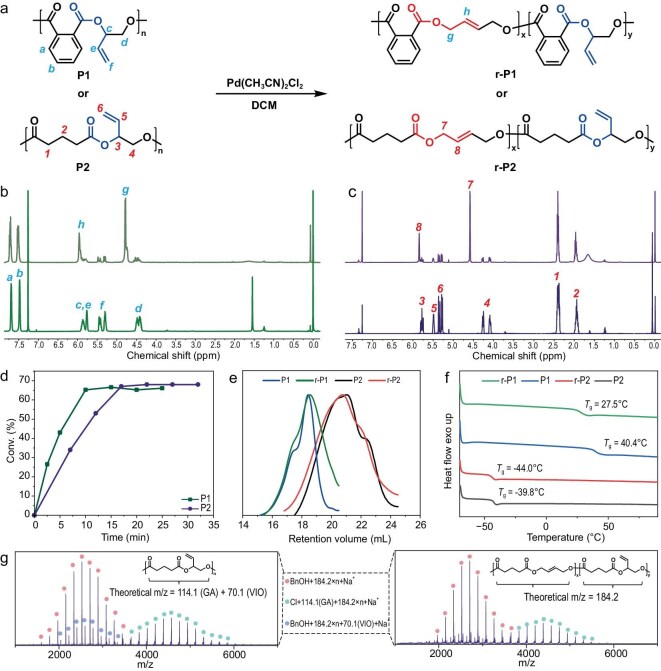
Characterization of the polymers and the rearranged polymers. (a) The rearrangement reaction of **P1** and **P2** catalyzed by Pd(CH_3_CN)_2_Cl_2_. (b) ^1^H NMR spectra for **P1** and **r-P1** (CDCl_3_). (c) ^1^H NMR spectra for **P2** and **r-P2** (CDCl_3_). See Supplementary data for details. (d) Plots of the equilibrium kinetics of the rearrangement **P1** to **r-P1** and **P2** to **r-P2**. (e) GPC traces of **P1**/**r-P1** and **P2**/**r-P2**. (f) DSC traces (second heating runs) of **P1**/**r-P1** and **P2**/**r-P2**. (g) MALDI-TOF analysis of **P2** (left) and **r-P2** (right).

With the polymers in hand, we then studied their backbone modification by classical [3,3]-sigma rearrangement condition. The synthesized polymer features a specific structure that is capable of undergoing [3,3]-sigma rearrangement under the [Pd] catalyst. The reaction proceeds through an acetoxonium intermediate, through which the terminal olefins would rearrange to *trans* internal ones (Fig. 2) [15,30,31]. For the purpose of screening for the optimal conditions, polyester **P1** (prepared from PA and VIO) was used for parallel rearrangement studies (Table 1, entry 1). We first employed Pd(CH_3_CN)_2_Cl_2_ as the catalyst under a loading of 2.5 mol%. The reaction was run for 1.5 h at room temperature under an inert atmosphere in dichloromethane (DCM, 0.1 M). As shown by ^1^H NMR comparing the raw materials and products, the signals at 5.95 and 4.78 ppm (the integral ratio was 1:2) (Fig. 3a and b) appeared for the rearranged counterpart’s *trans* protons. Meanwhile, the rearrangement product had a 72.3% yield (Table 1, entry 1). Moreover, as the rearrangement reactions were also unaffected by air (Table 1, entry 2), the reaction rates are similar (71.6%, Fig. S10). We then used tris(dibenzylideneacetone)dipalladium(0) [Pd_2_(DBA)_3_] [32] or Pd(OAc)_2_ [33] instead of Pd(CH_3_CN)_2_Cl_2_ to mediate the rearrangement. However, the rearrangement product did not appear (Table 1, entries 3 and 4). On the other hand, Pd(PPh_3_)_4_ [34,35] could also serve as a rearrangement catalyst with a lower conversion of 51.9% (Table 1, entry 5).

**Table 1.** Selected optimization experiments for rearranging **P1** and **P2**.

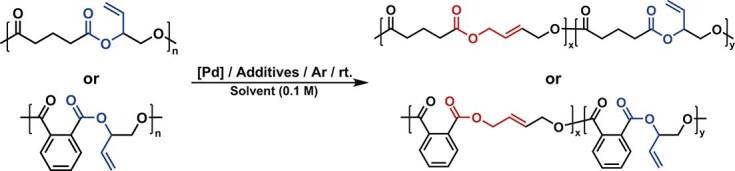						
Entry	Catalyst^a^	Additives^b^	Solvent	Time (h)	Conv. (P1)^c^ (%)	Conv. (P2)^c^ (%)
1	Pd(CH_3_CN)_2_Cl_2_		DCM	1.5	72.3	66.8
2^d^	Pd(CH_3_CN)_2_Cl_2_		DCM	1.5	71.6	67.3
3	Pd(OAc)_2_		DCM	4	0	0
4	Pd_2_(DBA)_3_		DCM	4	0	0
5	Pd(PPh_3_)_4_		DCM	4	51.9	54.5
6	Pd(CH_3_CN)_2_Cl_2_	TPP	DCM	4	0	63.7
7	Pd(CH_3_CN)_2_Cl_2_	dppf	DCM	4	0	2.8
8	Pd(CH_3_CN)_2_Cl_2_	RuPhos	DCM	4	0	5.0
9	Pd(CH_3_CN)_2_Cl_2_	AcOH	DCM	1.5	65.5	66.0
10	Pd(CH_3_CN)_2_Cl_2_	TEA	DCM	4	0	0
11	Pd(CH_3_CN)_2_Cl_2_		DMF	16	0	NA^e^
12	Pd(CH_3_CN)_2_Cl_2_		THF	4	67.5	57.5
13^f^	Pd(CH_3_CN)_2_Cl_2_			36	50.0	47.8

a [Pd] = 2.5 mol%. ^b^[Additives] = 5 mol%. ^c^Conversion rate (Conv.) determined by ^1^H NMR of purified polymers (CDCl_3_). ^d^Under air atmosphere. ^e^NA: not available. **P2** has a low solubility in DMF. ^f^Reaction temperature = 120°C.

To improve the rearrangement conversion, we then also incorporated ligands or additives that are widely used in organic synthesis catalyzed by the [Pd] system. In these cases, 1,1′-bis(diphenylphosphino)ferrocene (dppf) [36], 2-dicyclohexylphosphino-2′,6′-diisopropoxybiphenyl (RuPhos), triphenylphosphine (TPP) [34], triethylamine (TEA) [30] and anhydrous acetic acid (AcOH) [33] were also tested in the reaction system (Table 1, entries 6–10). After addition of AcOH, the rearrangement reaction could still proceed, while the conversion rate decreased slightly (Table 1, entry 9). However, the rearranged counterparts were not observed for other additives (Table 1, entries 6–8 and 10). It was therefore inferred that these additives could hinder the binding of the catalyst to the alkenyl groups, affecting the catalytic efficiency.

In addition, upon changing the solvent from DCM to *N,N*-dimethylformamide (DMF) [34], the reaction was also completely halted (Table 1, entry 11). Moreover, changing to tetrahydrofuran (THF) appeared to exert insignificant influence on the rearrangement (Table 1, entry 12), and the conversion rate would slightly decline to 67.5%. It is interesting to note that the rearranged counterparts could also be obtained under solvent-free conditions (Table 1, entry 13). However, the conversion rate was merely 50% owing to non-uniform diffusion, and polymer degradation was observed upon heating to 120°C (Fig. S11; PA was observed in ^1^H NMR).

Furthermore, the aliphatic polyester **P2** (prepared from GA and VIO) was also treated by the same conditions to verify the applicability of the rearrangement reaction. The results are similar to those of **P1** (Fig. 3a and c), and the addition of most additives also significantly affects the conversion of the rearrangement reaction. In contrast, addition of TPP had less effect on the rearrangement of **P2** (Table 1, entry 6), which might be because **P2** binds to [Pd] more quickly than TPP. Rearranged polymers can also be obtained when Pd(PPh_3_)_4_ is used as a catalyst, despite the presence of intensified side reactions including production of the *cis*-alkene structure (Fig. S12). Additionally, the rearrangement reaction was also not affected by air. Collectively, the rearrangement reaction appeared to only require the participation of a Pd catalyst; neither aromatic nor aliphatic anhydride-derived repeat units had a significant effect on the equilibrium conversion. Under the optimized conditions, **P1** could achieve a rearrangement conversion of 72.3% (Table 1, entry 1), and **P2** could achieve a conversion of 67.3% (Table 1, entry 2). The reaction kinetics also showed that the equilibrium of the rearrangement reaction would be reached within 20 mins (Fig. 3d, Figs S13 and S14). Since air had almost no influence on the rearrangement reaction, we chose the same conditions in Table 1, entry 2 as optimized condition to carry out all subsequent experiments for the sake of operational simplicity and obtaining structurally well-defined rearrangement products.

Further characterization of the polymers and rearranged polymers (re-polymers) was then conducted. Firstly, the rearranged alkenyl structure could be characterized by ^1^H NMR. In the case of **r-P2**, the signals at 5.78 and 4.52 ppm for the internal olefins (the integral ratio was 1:2; see Fig. S15 for details) appeared. Moreover, this result was further validated by heteronuclear singular quantum correlation (HSQC) (Fig. S16) and heteronuclear multiple quantum correlation (HMBC) spectroscopy (Fig. S17). As a reference, we also characterized **P2** using HSQC and HMBC spectroscopy (Figs S18 and S19). Based on the NMR results, **r-P2** possessed a well-defined *trans* alkenyl structure and no *cis* alkenyl structure was observed, which was consistent with the characteristic peak signals reported in the literature [37]. To further verify the presence of the *trans*-alkene structure in **r-P2**, we synthesized two oligomers via polycondensation of GA and *trans*-2-butene-1,4-diol (*trans*-BDO) or *cis*-2-butene-1,4-diol (*cis*-BDO). The signal of internal alkenes in **r-P2** overlapped with the alkene signal of the *trans* oligomer P[GA-*alt*-(*trans*-BDO)], and differed from the *cis* isomer P[GA-*alt*-(*cis*-BDO)]. This indicates that there is *trans*-alkene structure in **r-P2** with no *cis* structure (Fig. S20 and Fig. S21 for **r-P1**). As for the re-polymers, the molecular weight of repeat units is the same as their original polymers, and the rearrangement reaction does not affect their number-average molecular weight (*M*_n_). As shown in MALDI-TOF MS and GPC traces, the values of **r-P1** and **r-P2** were nearly identical to their origin polymers **P1** and **P2** (Fig. 3e and g, and Fig. S22). To demonstrate the broader utility of PBM, polyesters **P3**–**P6** and polycarbonate **P7** were reacted with Pd(CH_3_CN)_2_Cl_2_. Specifically, all reactions reached equilibrium within 1.5 h, and the rearrangement products were observed in all polymers, with polyester conversion exceeding 55.2% (Figs S23–S27). However, a conversion rate of only 27.5% was observed for polycarbonate **P7**.

**Figure 4.**
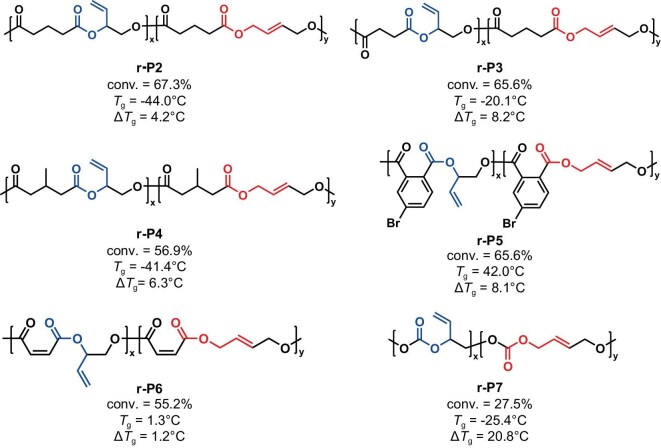
Re-polymers and their *T*_g_ values. The conversion rate (conv.) was calculated from ^1^H NMR of purified re-polymers.

**Figure 5.**
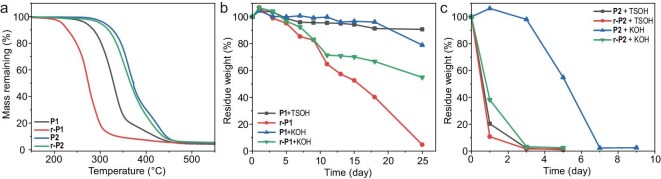
(a) TGA thermograms of polyesters **P1** and **P2** and their corresponding rearranged polymers **r-P1** and **r-P2** (10°C min^−1^). Mass loss profiles of polyesters (b) **P1**/**r-P1**, (c) **P2**/**r-P2** under 2 M TSOH (aq.) or 2 M KOH (aq.) degradation conditions at 60°C.

We then studied the thermal properties for the original and rearranged polymers. The re-polymers exhibited a reduced glass transition temperature (*T*_g_); the *T*_g_ of the re-polymer **r-P1** decreased from 40.4°C to 27.5°C (Δ*T*_g_ = 12.9°C), and no melting point was observed. Furthermore, similar decreases in *T*_g_ (4.2°C, 8.2°C, 6.3°C, 8.1°C, 1.2°C and 20.8°C) were observed for **P2**/**r-P2, P3**/**r-P3, P4**/**r-P4, P5**/**r-P5, P6**/**r-P6** and **P7**/**r-P7**, respectively (Figs 3f and 4, and Figs S28–S32). After dissolving in DCM, precipitating in EtOH and vacuum drying the samples three times to remove as much Pd impurities as possible, we performed thermal analysis on the polymers. The thermal degradation temperature at 5% mass loss (*T*_d,5%_) of **r-P1** (*T*_d,5%_ = 217.0°C) from thermogravimetric analysis (TGA) exhibited a substantial decline of 52.3°C compared with **P1** (*T*_d,5%_ = 269.3°C), whereas the decrease in **r-P2** (*T*_d,5%_ = 300.2°C) was 9.8°C to **P2** (*T*_d,5%_ = 310.0°C) (Fig. 5a). The reduction in the decomposition temperature of **P1** is significant. To further rule out the impact of [Pd] on the polymers, a series of control experiments were designed. First, we synthesized P(PA-*alt*-BO) by using PA and BO as monomers under the same conditions as the synthesis of **P1**, and the polyester was addressed by Pd(CH_3_CN)_2_Cl_2_. In this case, the rearrangement product r**-**P(PA-*alt*-BO) showed nearly identical *M*_n_, *T*_g_ (29.5°C/29.8°C) (Fig. S33) and *T*_d,5%_ (284.1°C/279.0°C) to its origin polyester (Fig. S34). Thereby, these results indicated that the [Pd] catalyst had no influence on the ester bond under the TGA conditions. Second, the rearrangement catalyst Pd(CH_3_CN)_2_Cl_2_ begins to decompose at 125°C (Fig. S35). The thermal degradation temperature of **P1** almost remains unchanged with or without additional [Pd] catalyst (Fig. S35). This indicates that the residual catalyst does not affect the degradation temperature under TGA conditions. Third, we used the polycondensation product P[PA-*alt*-(*trans*-BDO)] for TGA analysis as a comparison. The polycondensation products have a similar thermal degradation temperature to **r-P1** (Fig. S36). Furthermore, after directly mixing the polycondensation products with [Pd], their thermal degradation trends remain similar, even after observing the decomposition of [Pd]. This indicated that the decrease in the polymer’s decomposition temperature indeed originates from changes in the main chain structure. After ruling out the influence of residual [Pd], the *trans*-alkenyl structure of **r-P1** still has a lower thermal degradation temperature. This might be due to the *trans*-alkenyl structure reducing the steric hindrance for phthalate cyclization [38,39], leading to a lower decomposition temperature.

Polymers with internal alkene were assumed to have higher steric hindrance and lower reactivity compared with terminal alkene. To investigate the extent of reactivity during hydroboration–oxidation, we used 9-borabicyclo[3.3.1]nonane (9-BBN) to test the reactivity of polymers before and after modification. After feeding with 1.0 equiv. of 9-BBN to react at room temperature in an inert atmosphere for 1 h, an excess of 30% H_2_O_2_ aqueous solution was added for oxidation. It could be observed that the terminal alkenyl group in **r-P2** was almost completely converted (Fig. S37), while its internal alkene remains unchanged. Meanwhile, **P2** was converted by 80% (Fig. S38). If not treated by H_2_O_2_, both **r-P2** and **P2** would undergo cross-linking during the processes of sedimentation and desiccation into EtOH under air atmosphere [40]. Because of its lower density of terminal alkene, **r-P2** exhibited a lower cross-linking density (Fig. S39) and turned out in an oily state with a lower *T*_g_ of −44.6°C. For comparison, **P2** exhibited a higher cross-linking density (Fig. S39) and the product **P2** was a white solid with the absence of *T*_g_. This demonstrates that our rearrangement strategy also possesses potential for functional applications. These results demonstrate that the PBM strategy has great potential for accurate functionalization and controlled crosslinking of materials.

Another valuable feature of rearrangement is the introduction of alkenes into the polymer backbone that increases the chain flexibility and distance between ester linkages, which might increase degradation rates [39,41]. Therefore, it provides us with significant inspiration for our design regarding the lifecycle of polymers. The re-polyesters also have increased distance between ester linkages and chain flexibility compared with original polyesters. Thus, the re-polyesters would have higher degradability, which could provide a new method for polymer lifecycle modulation. To test our hypothesis, **P1, r-P1, P2** and **r-P2** were placed into a basic or acidic aqueous solution ([KOH] = 2 mol/L, [*p*-toluenesulfonic acid (TSOH)] = 2 mol/L) at 60°C to simulate the compost degradation process [42]. The remaining solids were filtered at the periodical degradation timepoints, then washed with water, vacuum dried and the remaining mass was weighed for analysis (Fig. 5b and c). At the beginning of the degradation process, the residue mass of almost all the samples increased in a small range. This may be owing to water uptake driven in the bulk erosion process [43]. Within 3 days, **P2** and **r-P2** were almost completely degraded (residue mass was less than 5%) when catalyzed by TSOH. Also, **r-P2** was also degraded obviously in KOH aqueous solution after 3 days with the mass of reduced solid decreased to 3%. In stark contrast, **P2** remained stable under the same conditions (Fig. 5c). For comparison, the degradation rates of **P1** and **r-P1** were slower; all the polyesters exhibited only minor degradation regardless of TSOH or KOH catalysis. This might be due to the poorer water solubility of degradation products or the hydrophobicity of the polymer chain [23]; not until 7 days did the notable degradation of **r-P1** begin. Subsequently, the remaining mass gradually declined over the course of time; however, 40% of the mass remained until the 18th day. For the longer periods, notable degradation (21% mass loss) was observed for **P1** in KOH on Day 25, while the mass loss of **P1** in TSOH still remained minimal. Additionally, ^1^H NMR analysis of the degradation products in KOH aqueous solution revealed that **P2** degraded into a mixture of corresponding diacids, polyols and other oligomers. In contrast, the ^1^H NMR spectrum of the degradation products of **r-P2** showed that the corresponding acids and other oligomers would be observed. Meanwhile, no evidence of *trans* polyols was present although there were still a few characteristic peaks of alcohols (Fig. S40). Subsequently, we also used liquid chromatography mass spectrometry (LC-MS) to detect the degradation products, which were diols and oligomers (Figs S41–S44 and Table S2 for **P2**; Figs S45–S47 and Table S3 for **r-P2**). This suggests that the structure of re-polymers may undergo further degradation into smaller molecules, which may promote enhancement of the degradation rates to a certain extent. Collectively, distinct lifecycles were established for the first time with backbone-editable polymers in this report.

## CONCLUSION

In conclusion, we synthesized a series of polymers via ROCOP of VIO and anhydrides/CO_2_, and introduced the rearrangement strategy into the synthesized polymers. The polymers contained special structures that can undergo rearrangement under the catalysis of [Pd], where the rearranged polymers would transform from terminal alkene to *trans* internal ones, with conversion range from 27.5% to 72.3%. This strategy assisted us to synthesize various polymers with new structures, which will offer new methods for the synthesis of new polyester materials. The rearranged polymers would exhibit subtle material property shifts. However, the re-polyesters showed a difference in reactivity with the original polymer; this holds significant implications for the accurate functional modification of polymers. Also, PBM in polyesters provides enhanced degradation in basic or acidic aqueous solutions. A series of synthesized polymers with complete degradation ranging from 3 to 25 days will offer opportunities for lifecycle design of polymers.

## Supplementary Material

nwaf534_Supplemental_File
