# Supplementary material for: Ring-opening copolymerization of 2-vinyloxirane with anhydride/carbon dioxide: facile access to backbone-editable polymers with tunable lifecycles
Source: Natl Sci Rev. 2025 Nov 28;12(12):nwaf534. doi: 10.1093/nsr/nwaf534 (PMC12728821; doi:10.1093/nsr/nwaf534)
Supplement: nwaf534_Supplemental_File [file nwaf534_supplemental_file.pdf]

Supplementary Information for

Ring-opening copolymerization of 2-vinyloxirane with anhydride/carbon dioxide: Facile access to backbone-editable polymers with tunable lifecycles

Mingxin Niu[1,2], Chenyang Hu[1,*], Zhenbiao Xie[1,2], Qi Zhang[1,2], Bokun Li[1,2], Zhiqiang Sun[1], Xuan Pang[1,2,*], Xuesi Chen[1,2]

[1]State Key Laboratory of Polymer Science and Technology, Changchun Institute of Applied Chemistry, Chinese Academy of Sciences, Changchun 130022, China;

[2]School of Applied Chemistry and Engineering, University of Science and Technology of China, Hefei 230026,

E-mails of corresponding authors: cyhu@ciac.ac.cn; xpang@ciac.ac.cn

Materials and Methods

1. Materials

Phthalic anhydride (PA), 4-bromophthalic anhydride (BPA), succinic anhydride (SA), glutaric anhydride (GA), maleic anhydride (MA), itaconic anhydride (IA), 3-methylglutaric anhydride (mGA), 1,2-epoxybutane (BO) are purchased from Innochem, 2-vinyloxirane (VIO) is purchased from Heowns Reagent, all the cyclic anhydrides are purified by recrystallization three times from ethyl acetate and dried under vacuum at room temperature before using, BO and VIO are distilled twice from CaH_2 under an argon atmosphere. Anhydrous acetic acid (AcOH), KOH, tetrahydrofuran (THF), triphenylphosphine (TPP), triethylamine (TEA) are purchased from Xiya Reagent. $Pd(OAc)_2$, $Pd(CH_3CN)_2Cl_2$, benzyl alcohol (BnOH), *p*-toluenesulfonic acid (TSOH), 9-borabicyclo[3.3.1]nonane (BBN), triethylborane (TEB, 1 mol/L in THF), anhydrous N,N-dimethylformamide (DMF), dimethyl sulfoxide (DMSO), anhydrous toluene (Tol), dichloromethane (DCM) are purchased from Energy Chemical. tris(dibenzylideneacetone)dipalladium(0) [$Pd_2(DBA)_3$], bis(triphenylphosphine)iminium chloride (PPNCl) are purchased from Sigma Aldrich. 1,1'-bis(diphenylphosphino)ferrocene (dppf), [1,2-cyclohexanediamino-N,N'-bis(3,5-di-t-butylsalicylidene)]chromium(III) chloride [(salen)CrCl], 2-dicyclohexylphosphino-2',6'-diisopropoxybiphenyl (RuPhos), *trans*-2-butene-1,4-diol (*trans*-BDO), *cis*-2-butene-1,4-diol (*cis*-BDO), glutaric acid are purchased from Bidepharm. All commercial reagents are used as received without further purification unless stated.

2. Structural Characterization

Nuclear magnetic resonance (^1H NMR) spectra were recorded on Bruker AV 500MHz in $CDCl_3$ at 25 °C unless stated. Chemical shifts were given in parts per million from tetramethylsilane. Gel permeation chromatography (GPC) measurements were conducted with a Waters 515 GPC with CH_2Cl_2 as the solvent (flow rate: 1 mL/min, at 30 °C), the molecular weights were calibrated against polystyrene (M_n = 1250–332,800 kDa) standards. Polymer samples were analysed on a DSC25 (TA Instruments) under a N_2 flow (50 mL/min). Samples were heated (10 °C/min) and equilibrated to 100 ~ 200 °C to remove their thermal history, then cooled to -70 °C (10 °C/min) before heating a second time to 100 ~ 200 °C at a rate of 10 °C/min, The TGA was carried out using a TA TGA5500 (TA Instruments) analyses, characterization was performed in N_2 flow (50 mL/min) using platinum pans with a heating rate of 10 °C/min from 50 °C to 600 °C. MALDI-TOF MS experiments were carried out on Waters/Micromass MALDI micro MX spectrometer, using a dithranol matrix in EA or chloroform at loading of 1:1 with potassium trifluoroacetate (KO_2CCF_3) as the cationizing agent. Liquid Chromatography Mass Spectrometry (LC-MS) analysis was carried out on Waters LCT Premier bench-top orthogonal acceleration time-of-flight LC-MS system.

3. General polymerization procedure

All polymerizations were carried out in a dry argon atmosphere using standard Schlenk techniques or in a glovebox. In a typical polymerization experiment, cyclic anhydride was loaded in a flame-dried vial containing a magnetic bar, and catalytic system, initiator and epoxides in desired ratios were added, under some conditions, a certain solvent should be added. The vial was sealed under argon, and placed in an oil bath thermostated at 80 °C or 60 °C. After a certain reaction time, minimal amount of sample was withdrawn for ^{1}H NMR analysis. The polymers were isolated by precipitating into ethanol. The precipitate was collected and dried under vacuum at 50 °C overnight [1-3].

For the co-polymerization of CO_2 and VIO, in a glovebox, TEB, PPNCl, initiator and VIO in desired ratios were added into a flame-dried autoclave containing a magnetic bar, and the autoclave was taken out of glovebox, then the pressure of CO_2 is elevated to 2 MPa via the high-pressure CO_2 cylinder. The reaction was placed in an oil bath thermostated at 80 °C. After a certain reaction time, minimal amount of sample was withdrawn for ^{1}H NMR analysis. The polymers were isolated by precipitating into ethanol. The precipitate was collected and dried under vacuum at 50 °C overnight[4, 5].

4. General procedure of rearrangement reaction

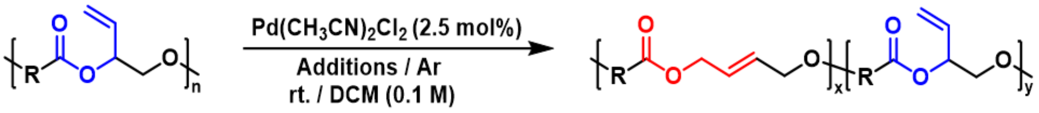

In a glove box with nitrogen atmosphere, to an oven-dried 20 mL Schlenk flask with a stir bar was added **P1** (0.1015 g) corresponding addition and solvent (0.1 M), then $Pd(CH_3CN)_2Cl_2$ (3.0 mg, 2.5 mol%) was added after **P1** completely dissolved. The vial was then taken out of the glovebox. The reaction mixture was stirred at room temperature for 1.5 hours. The reaction mixture was quenched by DMSO, and poured into ethanol, the solid was then collected by centrifuging, then dried under vacuum at 50 °C overnight until constant weight [6-10].

5. Reaction of P2 or r-P2 with BBN

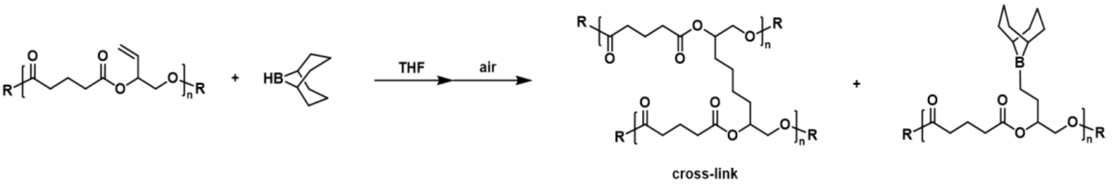

In a glove box with nitrogen atmosphere, to an oven-dried 20 mL Schlenk flask with a stir bar was added **P2** (0.0510 g) or **r-P2** (0.0510 g), then 1 equiv. BBN solution (0.5 M in THF，0.55 mL) was added. The vial was then taken out of the glovebox. The reaction mixture was stirred at room temperature for 1 hour. Then add 0.5 mL of 30% H_2O_2 aqueous solution in an inert gas atmosphere and continue stirring for 30 minutes. Then the solid was filtered, washed with water three times, dried under vacuum at 50 °C overnight until constant weight.

As for the crosslink, the reaction mixture was stirred at room temperature for 4 hour after the system was taken out of the glovebox, and then poured into ethanol, the solid was then collected by centrifuging, then dried under vacuum at 50 °C overnight until constant weight [3, 11].

6. General polymerization procedure

All the syntheses and manipulations of the polyesters were carried out in an over-dried 250-mL three-necked round-bottom flasks equipped with a magnetic bar. In a typical polymerization experiment, the flask was charged with the diol and excess diacid (1.1 equiv). In the first esterification stage, the reaction was performed at 200 °C for 4 h under an argon flow to completely remove the water. In the second polymerization stage, after turning off the argon flow, the flask was connected to a cold trap (liquid nitrogen) and then a vacuum pump. The reaction was further carried out at 240-280 °C and under high vacuum (<100 Pa) for 4 h. [12]

7. General procedure of degradation under basic or acidic aqueous solution

To a Schott bottle charged with **P1**, **P2**, **r-P1**, **r-P2** (0.100g) was separately added an aqueous solution of KOH (2 M, 3 mL) or TSOH (2 M, 3 mL). The suspension was sealed, heated to 60 °C and stirred using a magnetic stirrer bar, at 1000 rpm. At the desired day, the polymer samples were taken out of solution, washed with a large amount of water and dried, and then weighed the residue mass. Then the samples were placed into their origin solutions. In the case of all samples a completely homogeneous solution was observed. ^{1}H NMR analysis: After completely degradation, the solution was quenched until pH = 7, a minimal quantity of the solution was subjected to concentrating, followed by the addition of D_2O as a solvent ^{1}H NMR) analysis [13].

Supplementary Figures and Tables

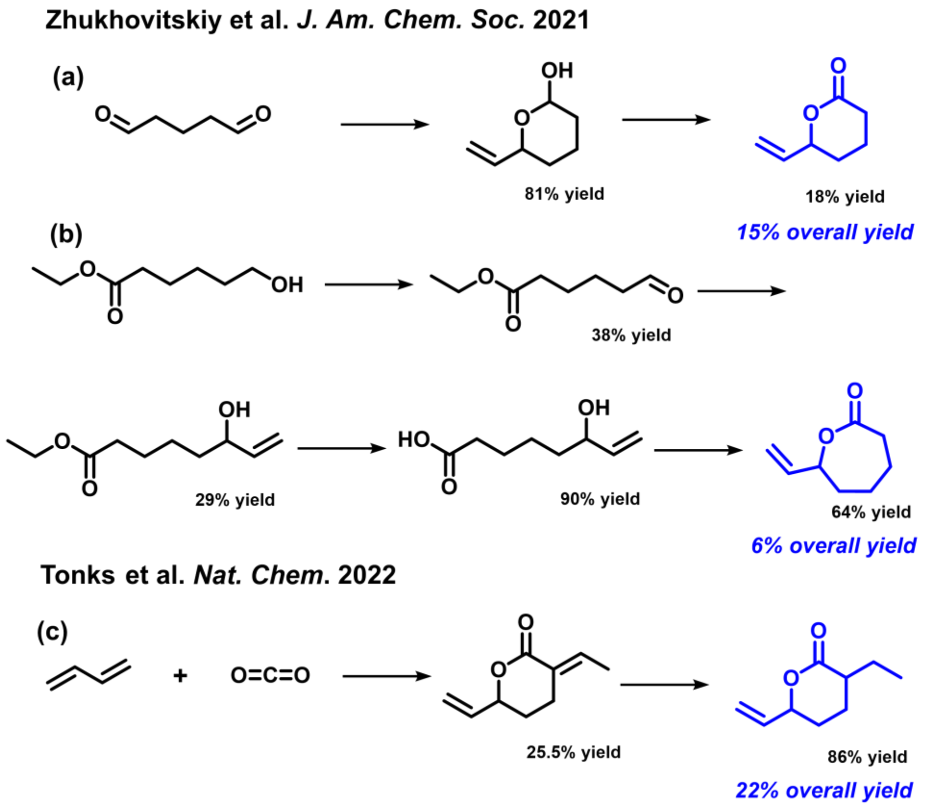

Figure S1. The synthetic route of ω-vinyllactones in previous works [14, 15].

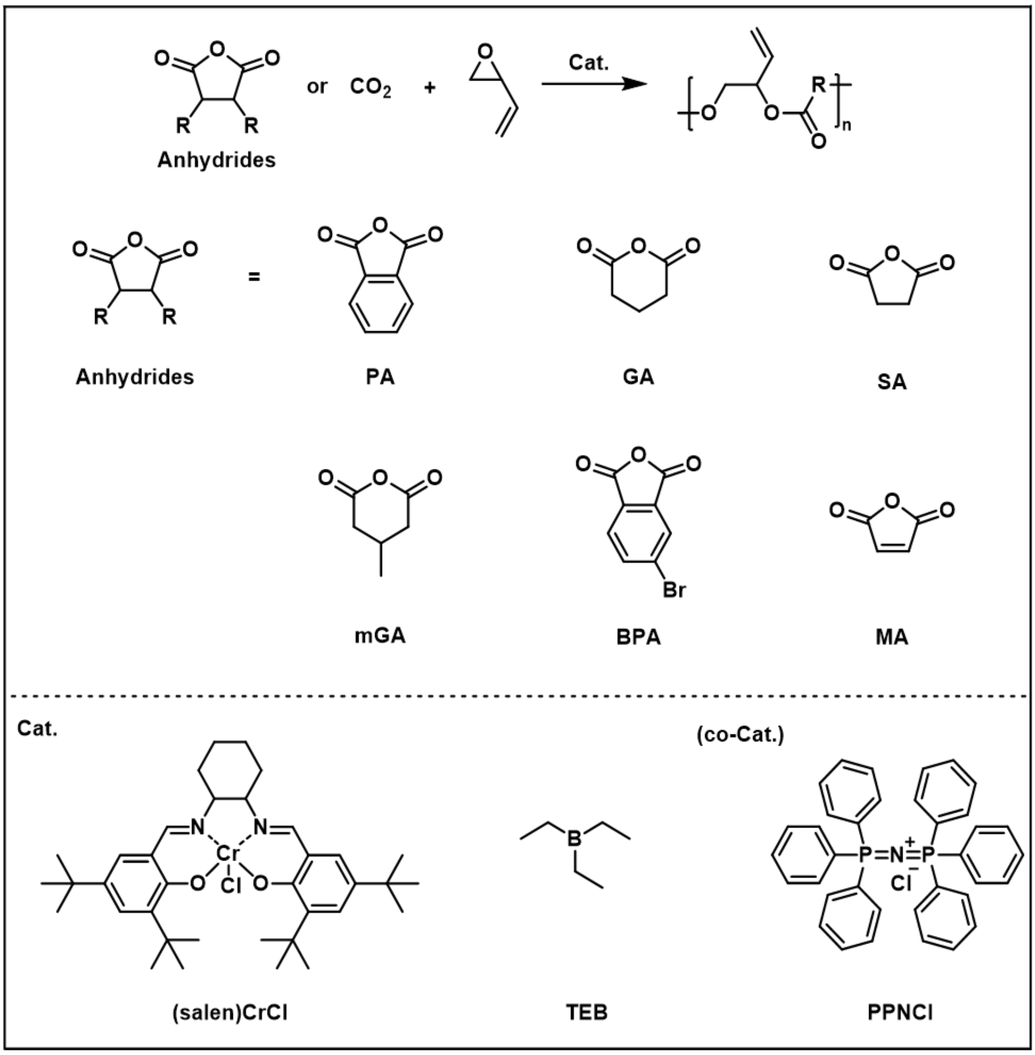

Table S1. Copolymerization of VIO and cyclic anhydrides or CO_2

Entry	Monomer	Cat.	[M]/[VIO]/ [Cat.]/[co-Cat.]	*T* / °C	Time / h	Conv. / %[a]	M_n / kDa[b]	*Đ*[b]
1(**P1**)	PA	(salen)CrCl/PPNCl	400:600:1:1	80	8	> 99	9.8	1.34
2(**P2**)	GA	(salen)CrCl/PPNCl	300:450:1:1	80	18	> 99	4.6	1.52
3(**P3**)	SA	(salen)CrCl/PPNCl	200:300:1:1	60	18	> 99	3.5	1.50
4(**P4**)	mGA	(salen)CrCl/PPNCl	300:450:1:1	80	18	> 99	6.9	1.44
5(**P5**)	MA	(salen)CrCl/PPNCl	300:450:1:1	60	16	21.5	1.5	1.65
6(**P6**)	BPA	(salen)CrCl/PPNCl	200:300:1:1	80	8	> 99	5.7	1.53
7(**P1**)	PA	TEB/PPNCl	200:300:1.5:1[c]	80	8	> 99	6.5	1.15
8(**P2**)	GA	TEB/PPNCl	200:300:1.5:1[c]	80	18	> 99	6.9	1.51
9(**P7**)	CO_2	TEB/PPNCl	400:2:1[d]	80	96	43.9	15.5	1.34

[a]Calculated from ^{1}H NMR of crude reaction aliquot. [b]Determined by gel permeation chromatography (GPC) calibrated with polystyrene standards in CH_2Cl_2 at 30 °C. [c]BnOH as initiator was added (BnOH/TEB/PPNCl = 5/1.5/1, all in molar ratio). [d] The press of CO_2 was 2.0 MPa, and BnOH as initiator was added (BnOH/PPNCl = 2/1).

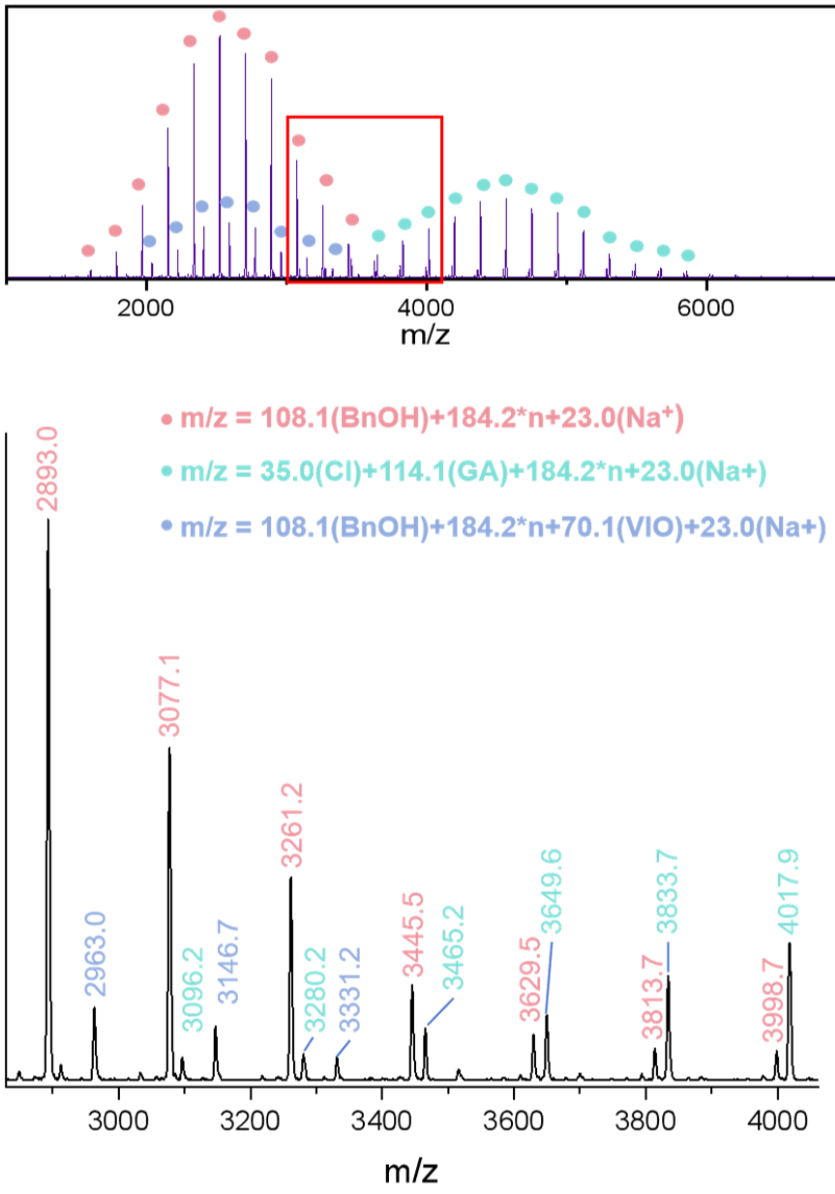

Figure S2. MALDI-TOF analysis of **P2** (Table S1, entry 8).

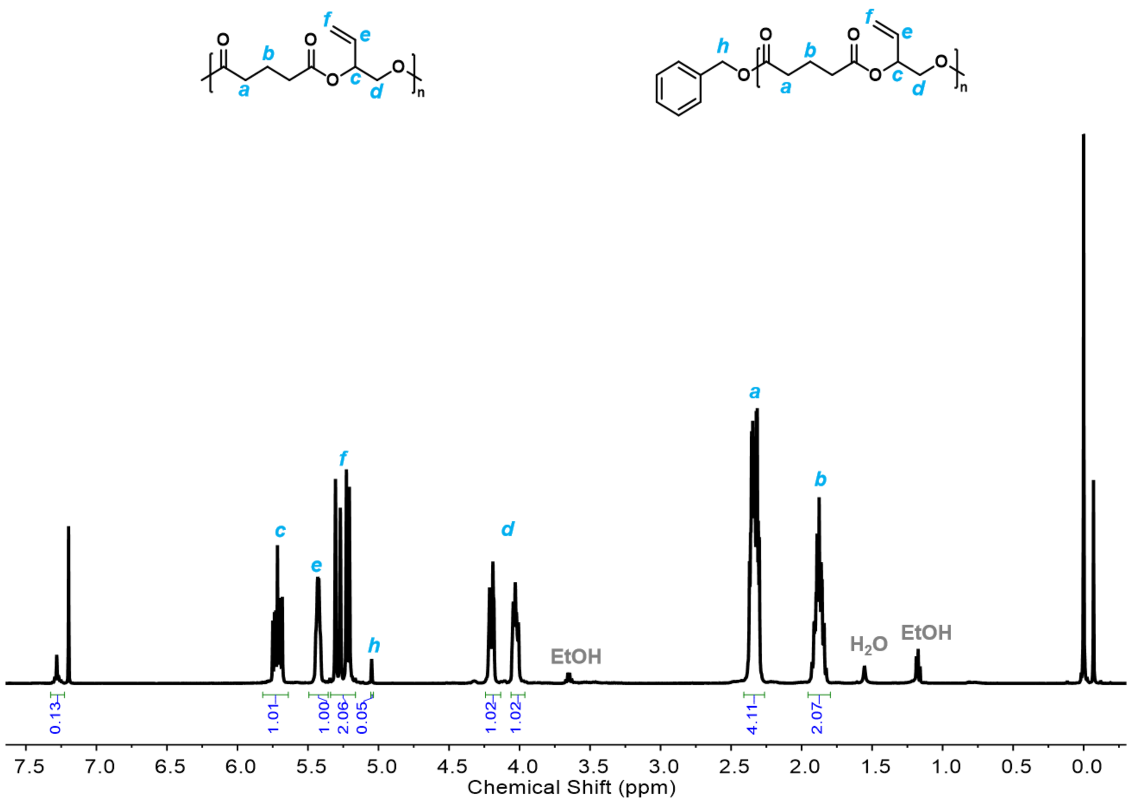

Figure S3. ^{1}H NMR spectrum for polyester **P2** ($CDCl_3$) (Table S1, entry 8).

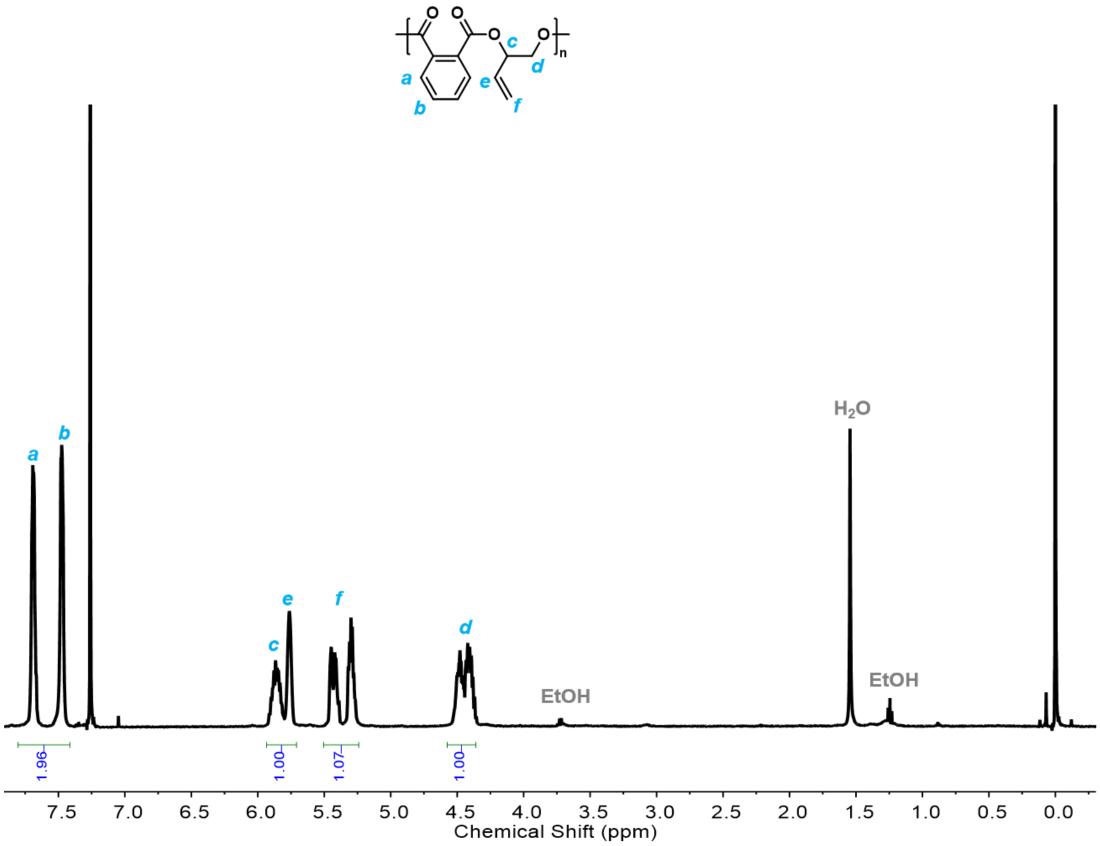

Figure S4. ^{1}H NMR spectrum for polyester **P1** ($CDCl_3$).

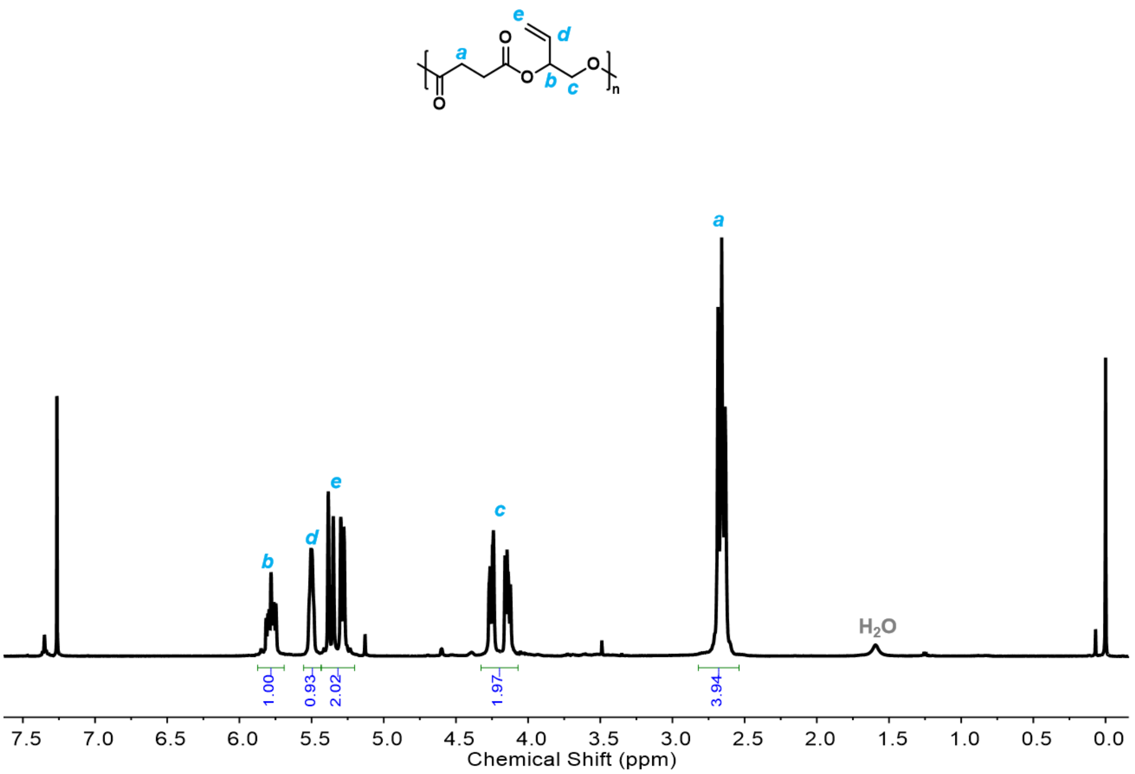

Figure S5. ^{1}H NMR spectrum for polyester **P3** ($CDCl_3$).

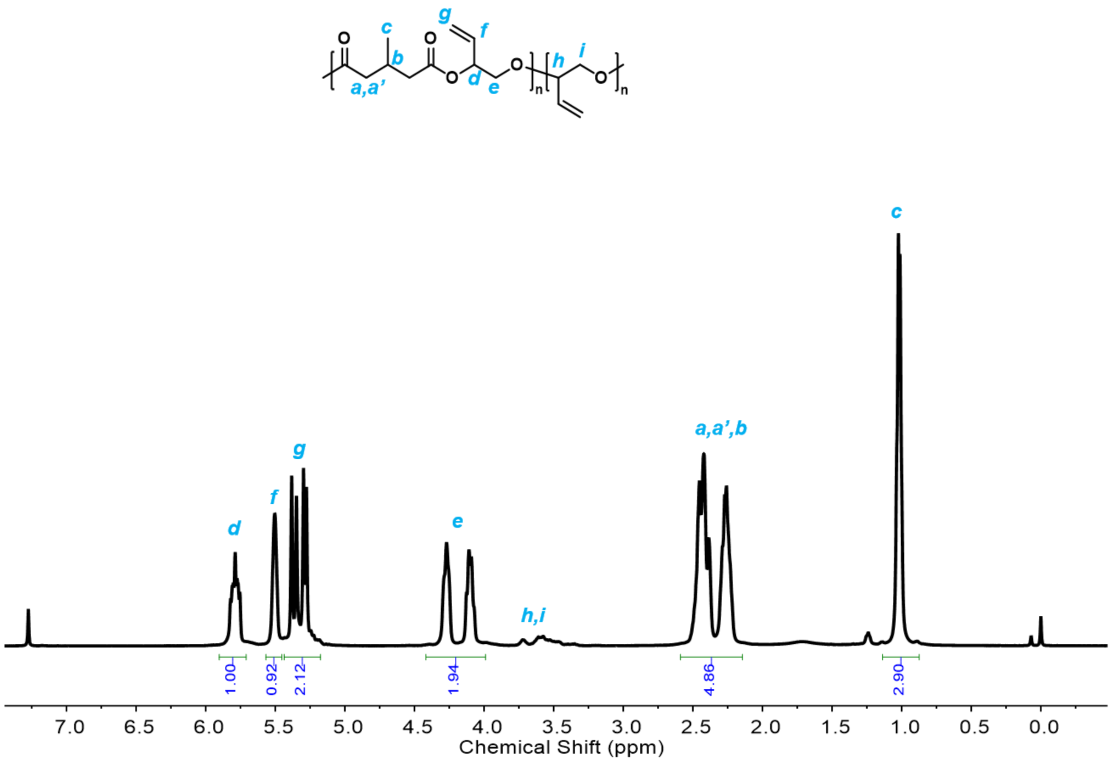

Figure S6. ^{1}H NMR spectrum for polyester **P4** ($CDCl_3$).

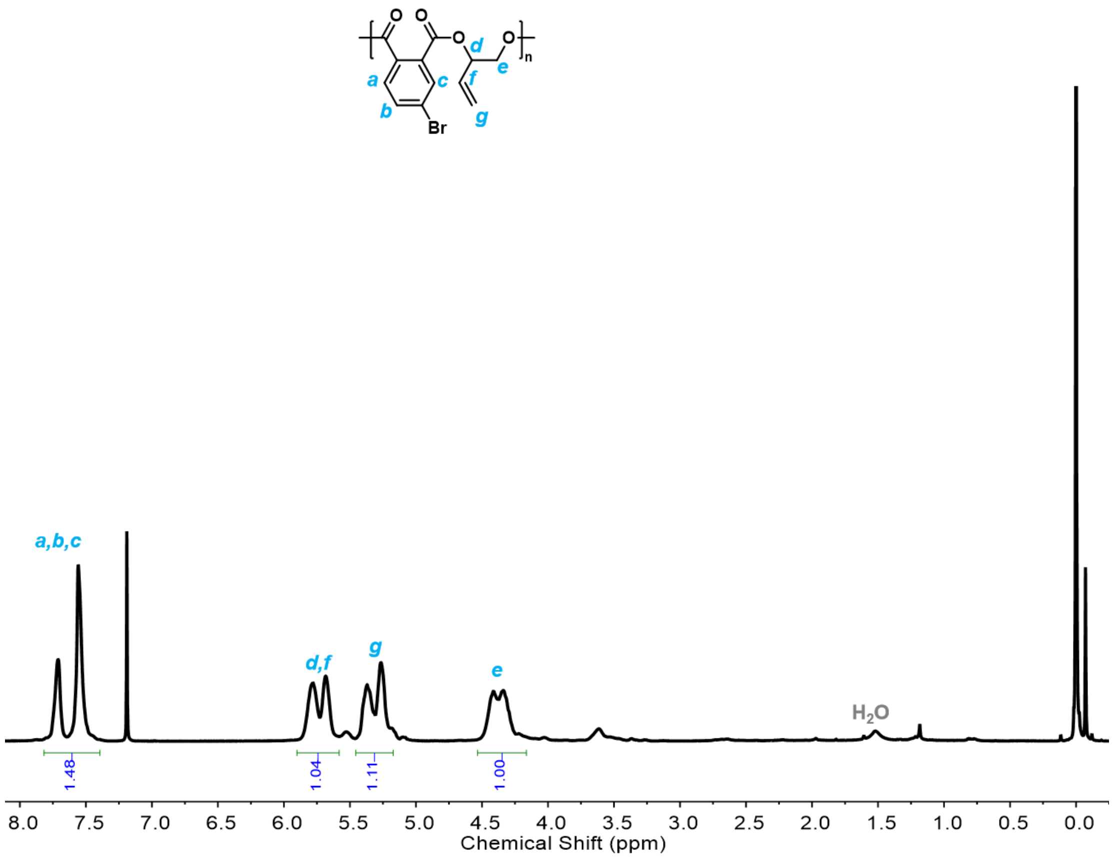

Figure S7. ^{1}H NMR spectrum for polyester **P5** ($CDCl_3$).

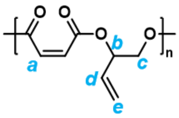

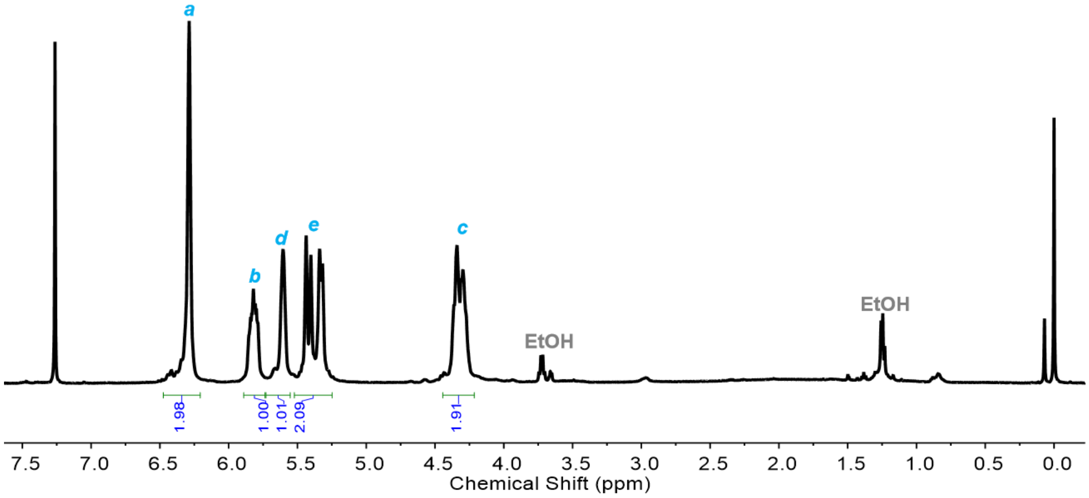

Figure S8. ^{1}H NMR spectrum for polyester **P6** ($CDCl_3$).

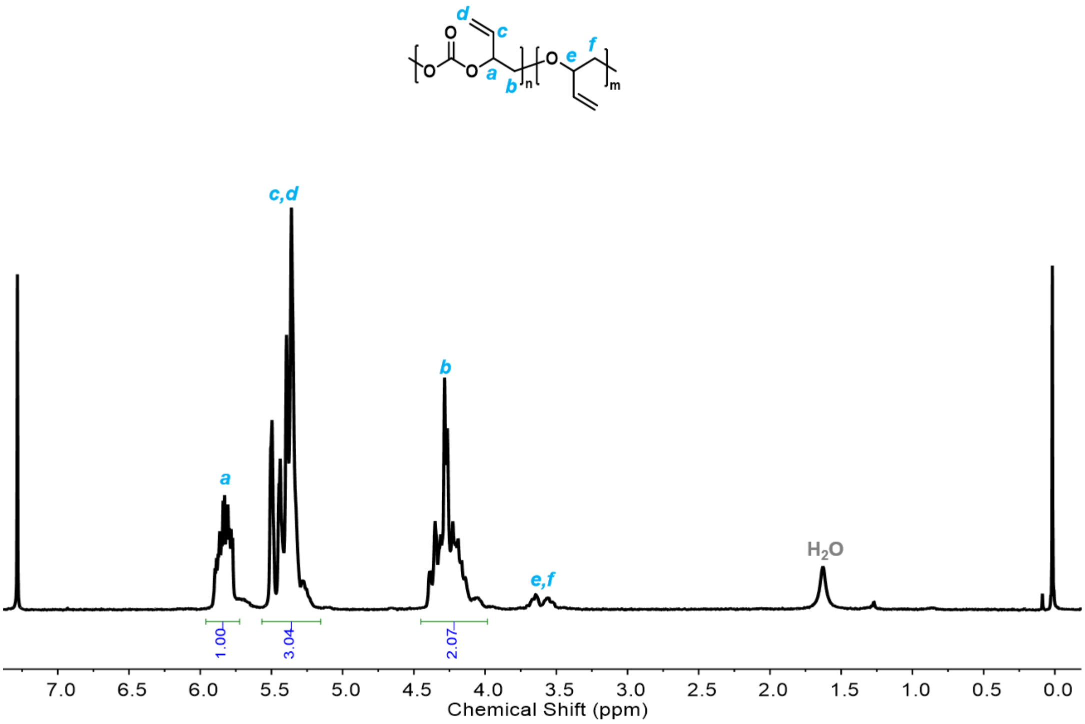

Figure S9. ¹H NMR spectrum for polyester **P7** ($CDCl_3$).

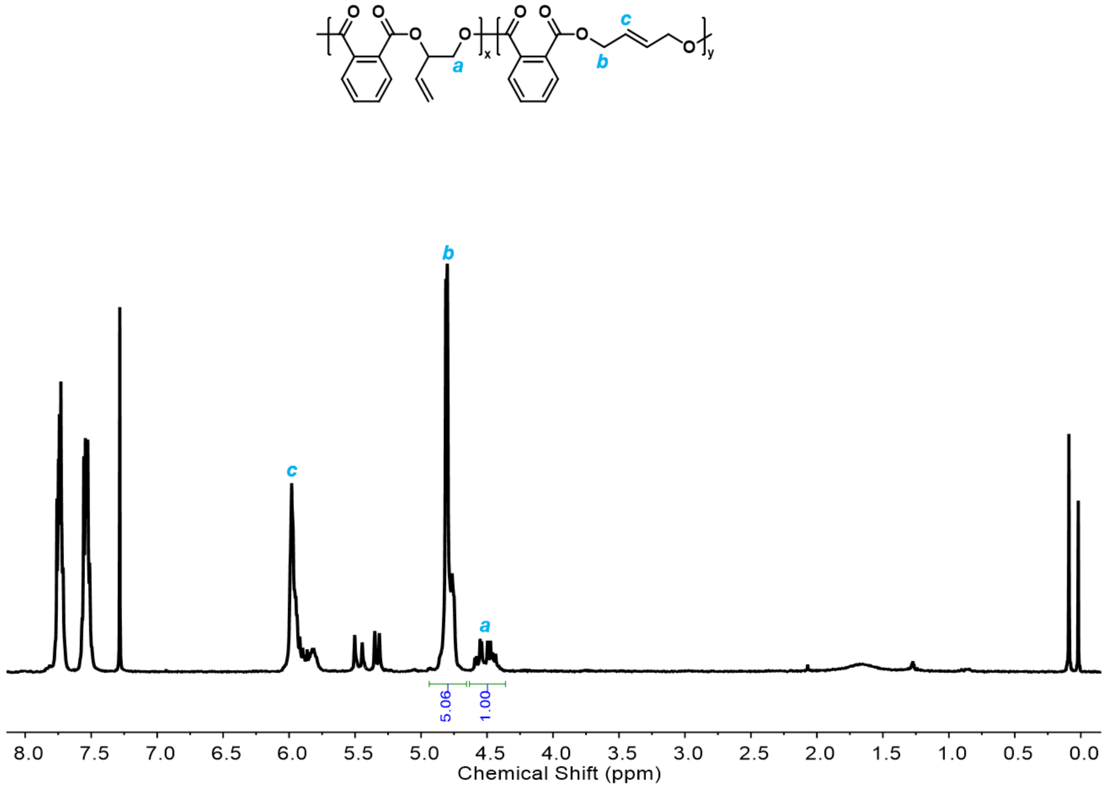

Figure S10. ^{1}H NMR spectrum for polyester **r-P1** ($CDCl_3$). The conversion rate of [3,3]-sigmatropic oxo-rearrangement was 71.6%. The rearrangement conversion = H_b / (H_b + 2H_a) * 100% = 71.6%, H_a and H_b are the integral ratios of peaks a and b, respectively.

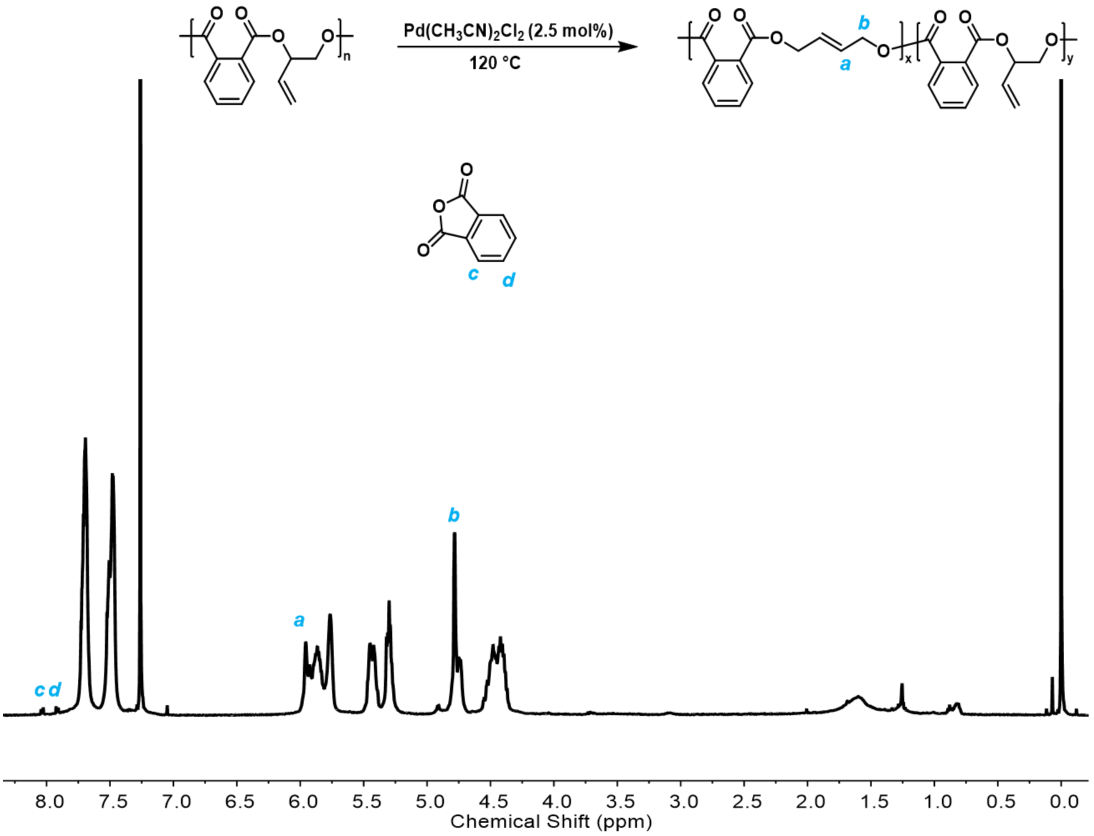

Figure S11. ^{1}H NMR spectrum for rearranged polyester **P1** ($CDCl_3$), phthalic anhydride was observed. Reaction conditions: 120°C, [Pd] = 2.5 mol%, solvent free.

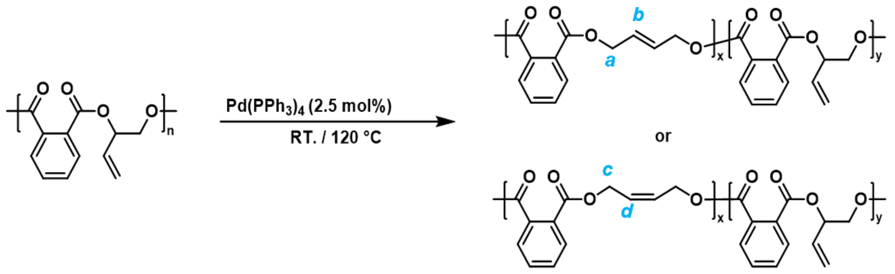

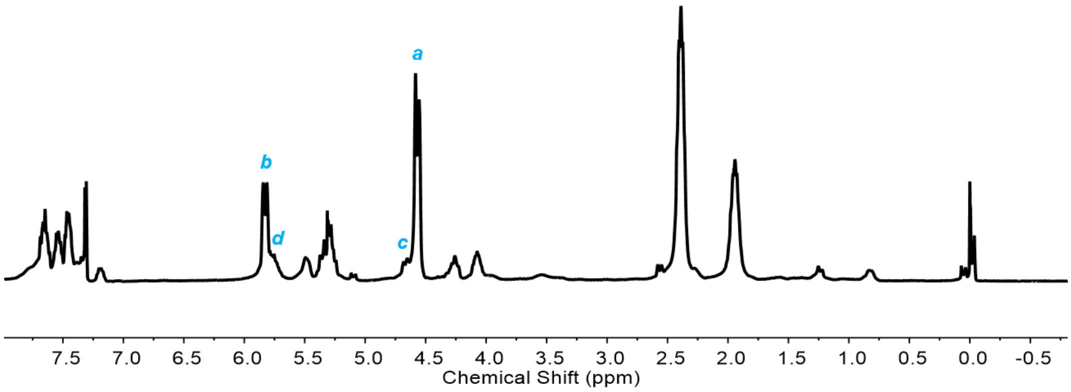

Figure S12. ^{1}H NMR spectrum for rearranged polyester **P2** ($CDCl_3$), *trans*-alkene structure was observed. Reaction conditions: Room temperature, [Pd(PPh_3)$_4$] = 2.5 mol%, DCM.

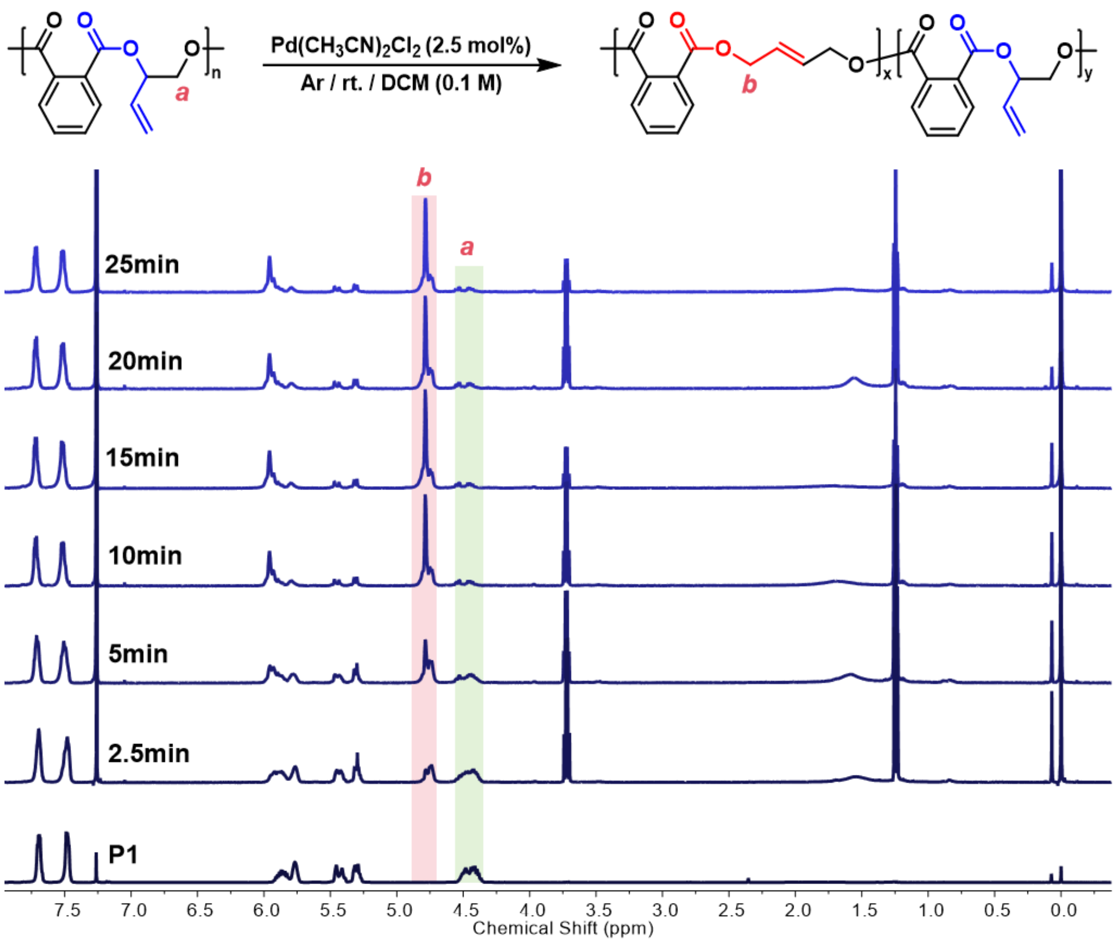

Figure S13. ^{1}H NMR at various times throughout the rearrangement reaction of **P1** with $Pd(CH_3CN)_2Cl_2$ at 20°C.

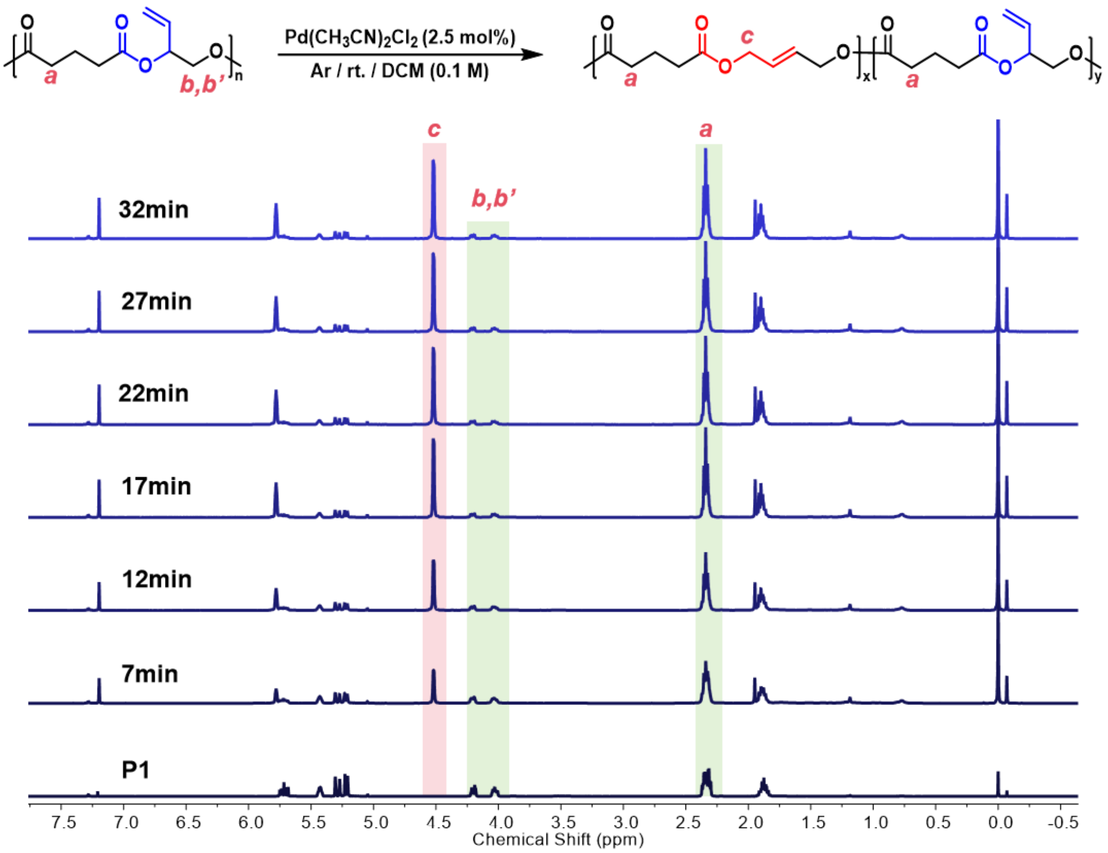

Figure S14. ^{1}H NMR at various times throughout the rearrangement reaction of **P2** with $Pd(CH_3CN)_2Cl_2$ at 20°C.

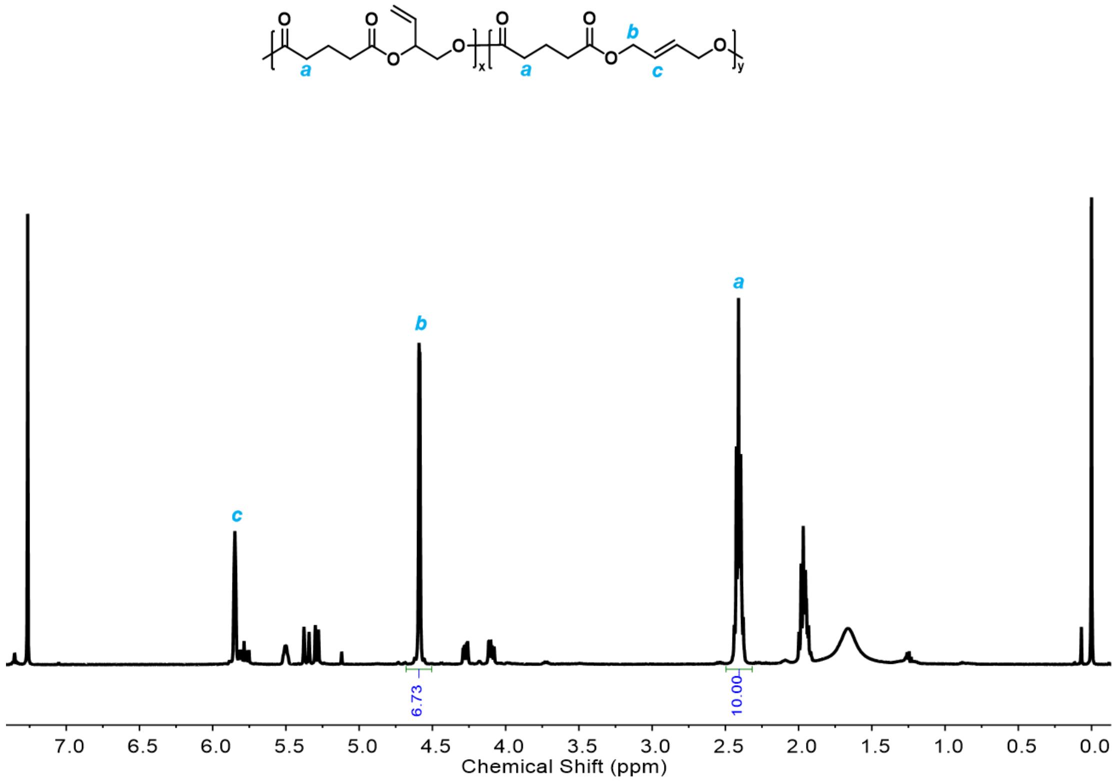

Figure S15. ^{1}H NMR spectrum for polyester **r-P2** ($CDCl_3$). The conversion rate of [3,3]-sigmatropic oxo-rearrangement was 67.3%. The rearrangement conversion = H_b / H_a * 100% = 67.3%, H_a and H_b are the integral ratios of peaks a and b, respectively.

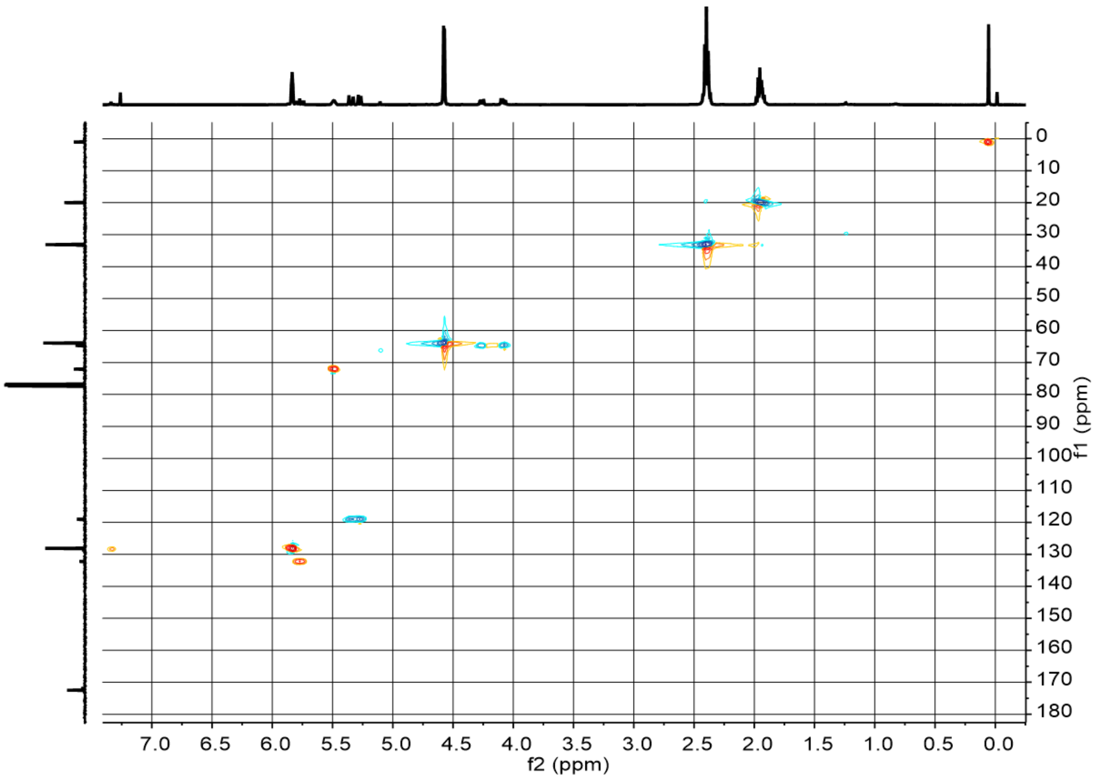

Figure S16. Heteronuclear singular quantum correlation (HSQC) NMR (600 MHz, $CDCl_3$, 23°C) spectrum of polyester **r-P2**.

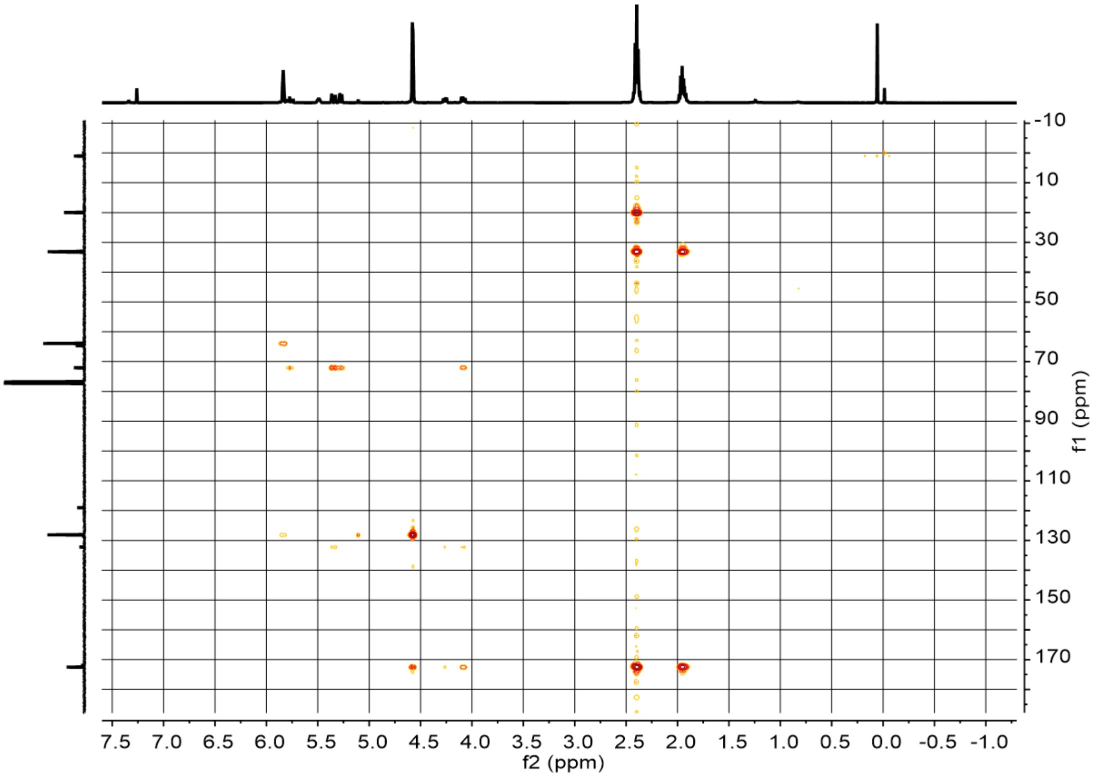

Figure S17. Heteronuclear multiple quantum correlation spectroscopy (HMBC) NMR (600 MHz, $CDCl_3$, 23°C) spectrum of polyester **r-P2**.

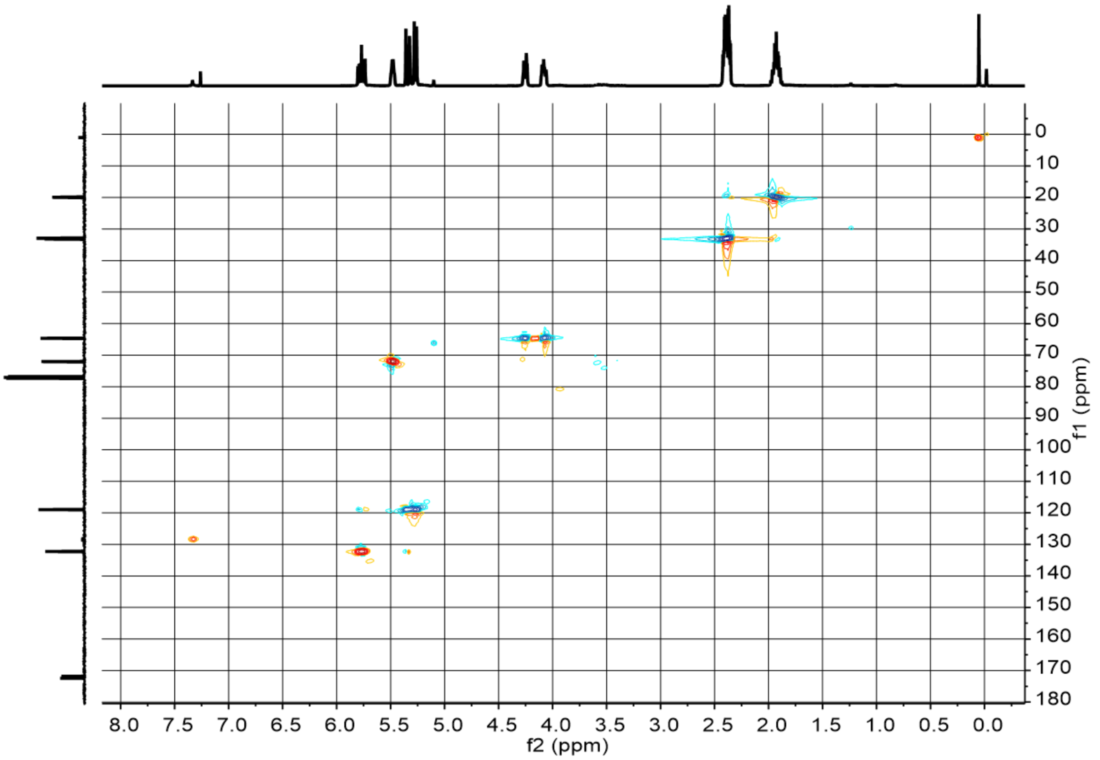

Figure S18. Heteronuclear singular quantum correlation (HSQC) NMR (600 MHz, $CDCl_3$, 23 °C) spectrum of polyester **P2**.

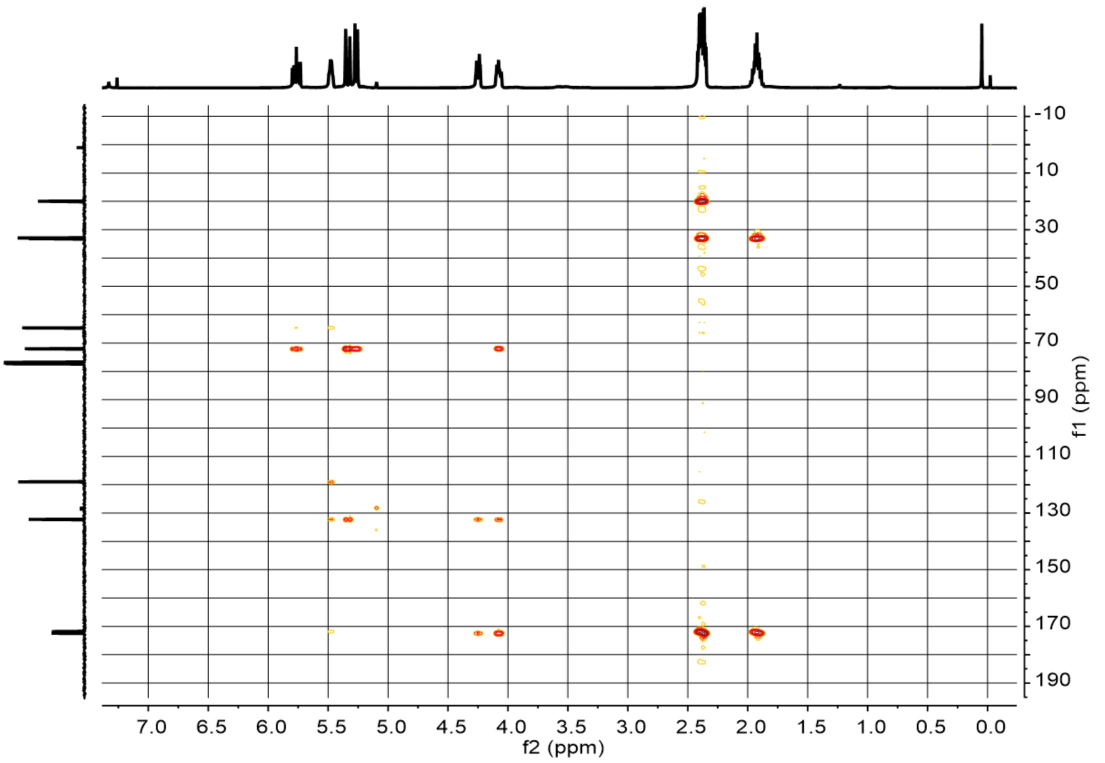

Figure S19. Heteronuclear multiple quantum correlation spectroscopy (HMBC) NMR (600 MHz, $CDCl_3$, 23°C) spectrum of polyester **P2**.

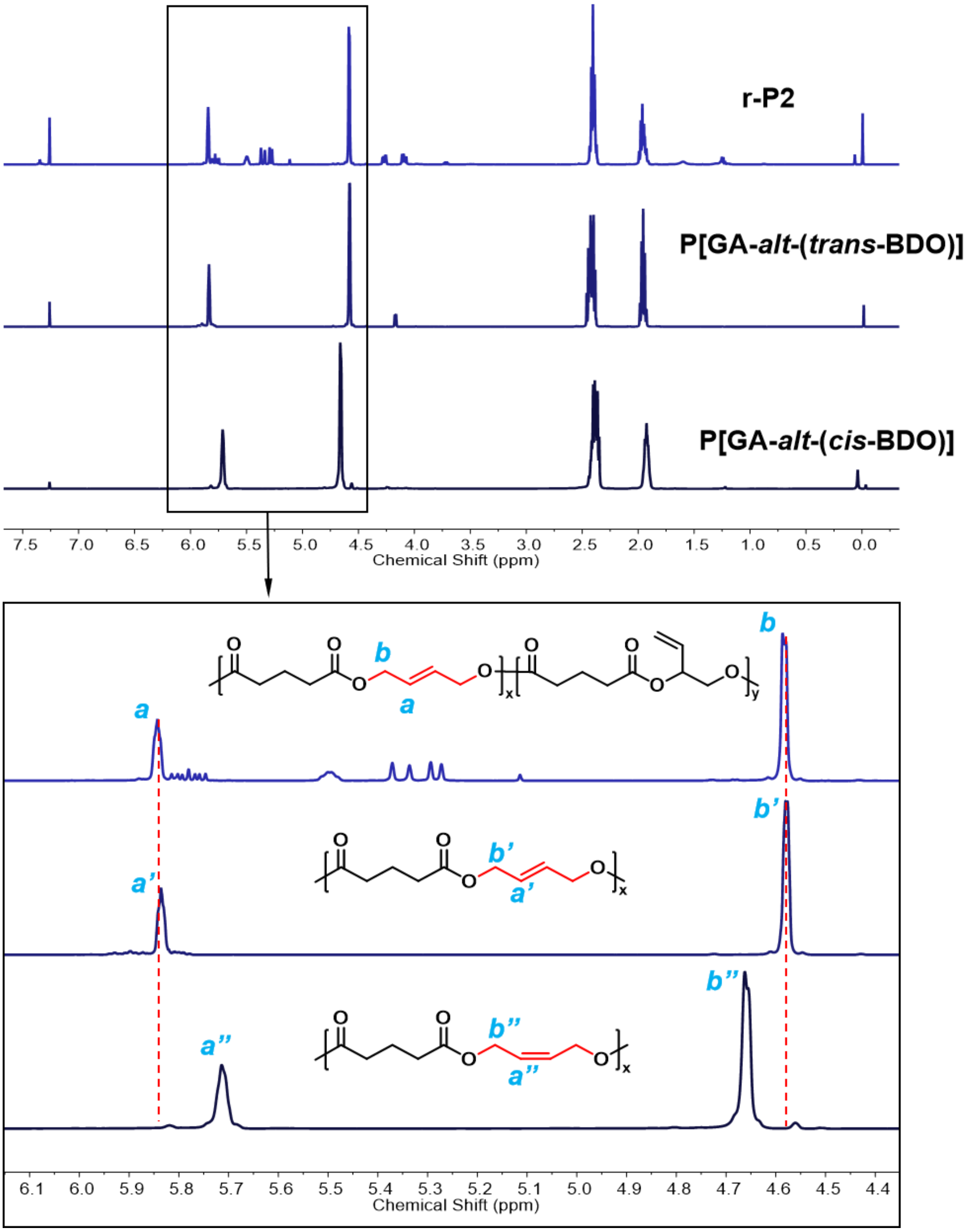

Figure S20. ^{1}H NMR spectrum (CDCl3) for polyester **r-P2,** P[GA-*alt*-(*trans*-BDO)] (prepared from polycondensation of GA and *trans*-BDO)**,** P[GA-*alt*-(*cis*-BDO)] (prepared from polycondensation of GA and *cis*-BDO).

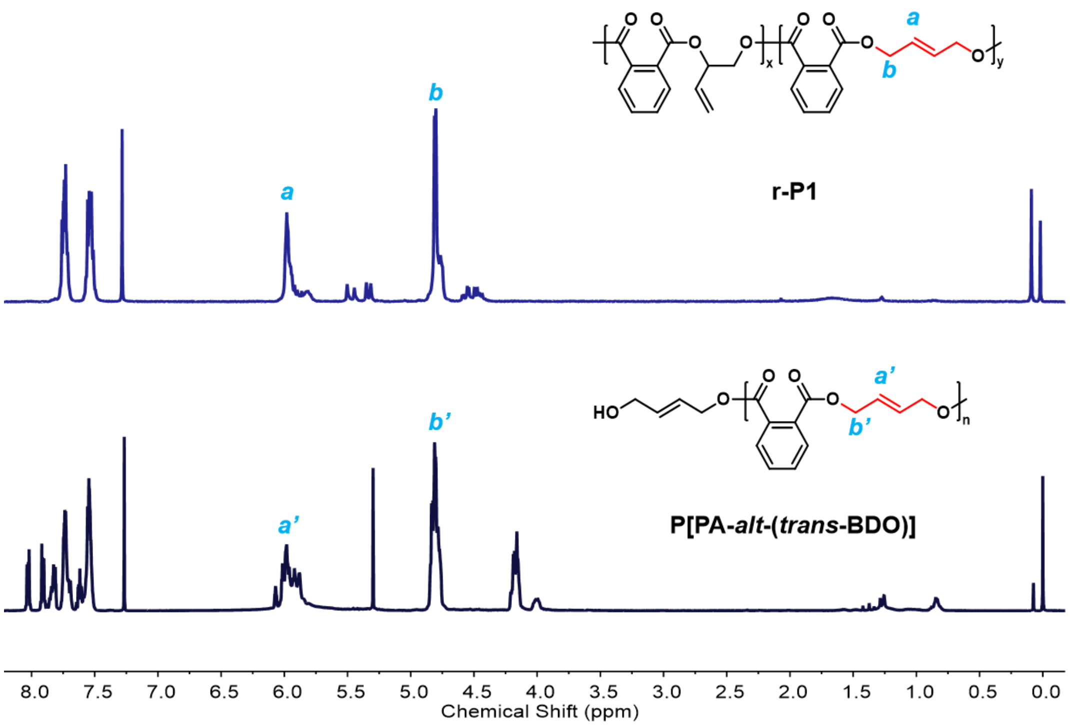

Figure S21. ^{1}H NMR spectrum ($CDCl_3$) for polyester **r-P1** and P[PA-*alt*-(*trans*-BDO)] (prepared from polycondensation of (PA and *trans*-BDO).

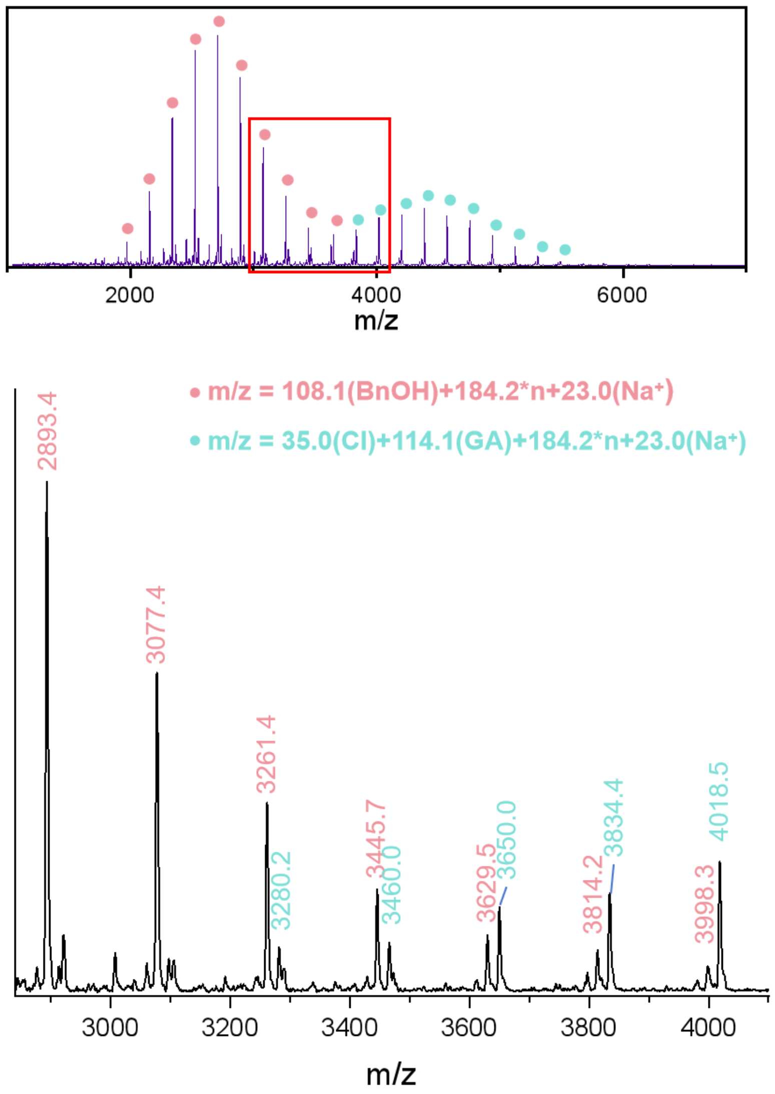

Figure S22. MALDI-TOF analysis of **r-P2**.

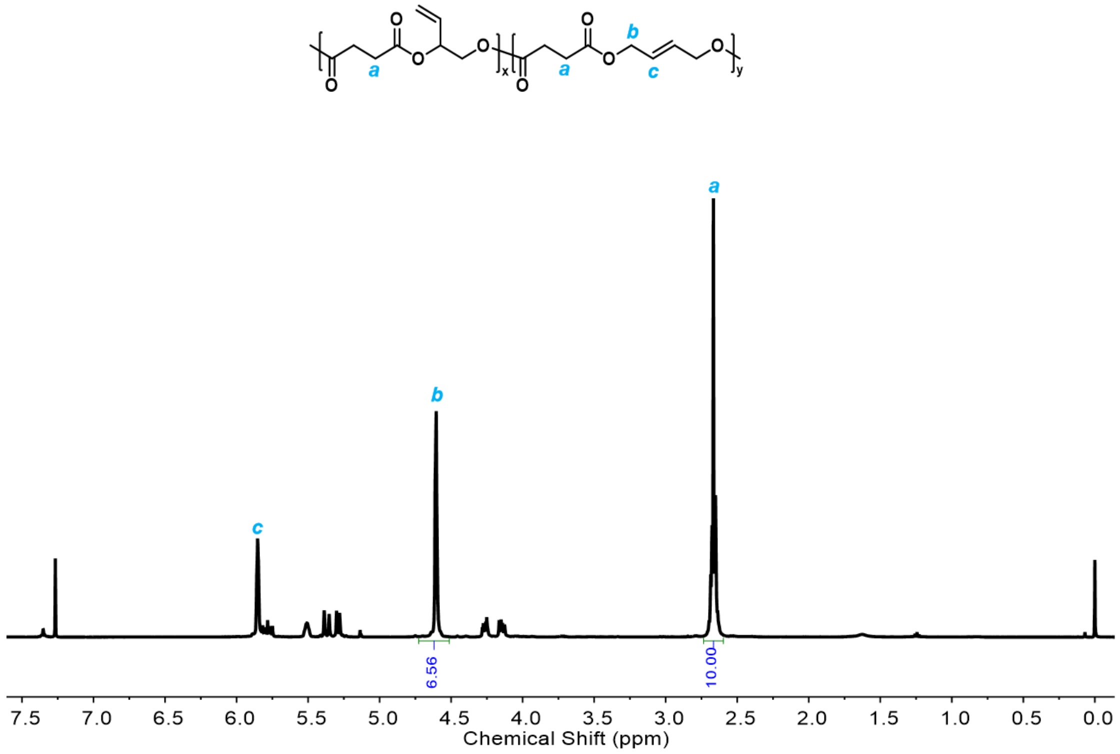

Figure S23. ^{1}H NMR spectrum for polyester **r-P3** ($CDCl_3$). The conversion rate of [3,3]-sigmatropic oxo-rearrangement was 65.6%. The rearrangement conversion = H_b / H_a * 100% = 65.6%, H_a and H_b are the integral ratios of peaks a and b, respectively.

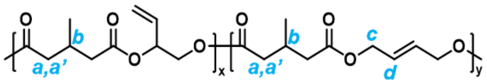

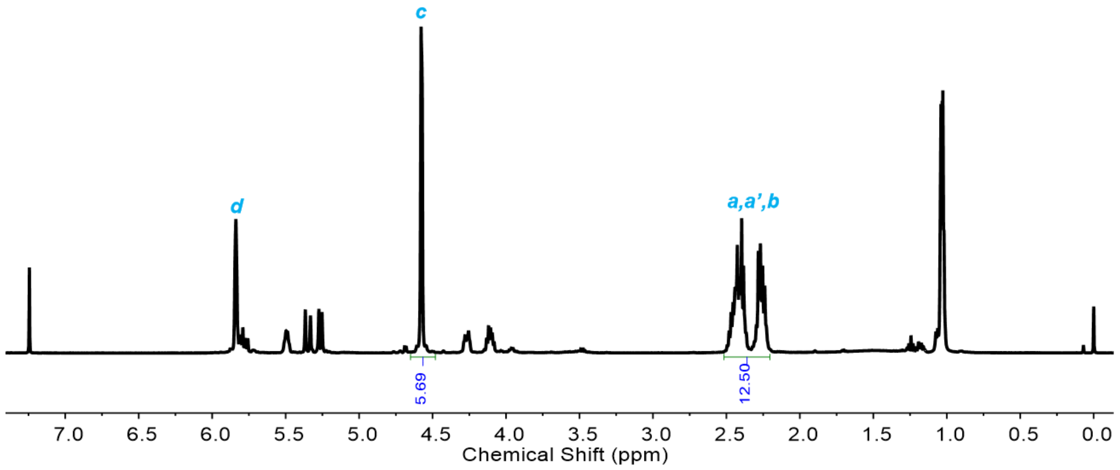

Figure S24. ^{1}H NMR spectrum for polyester **r-P4** ($CDCl_3$). The conversion rate of [3,3]-sigmatropic oxo-rearrangement was 56.9%. The rearrangement conversion = H_c / [0.8 * (H_a + $H_{a'}$ + H_b) + H_c] * 100% = 56.9%, H_a, $H_{a'}$, H_b and H_c are the integral ratios of peaks a, a', b and c peaks, respectively.

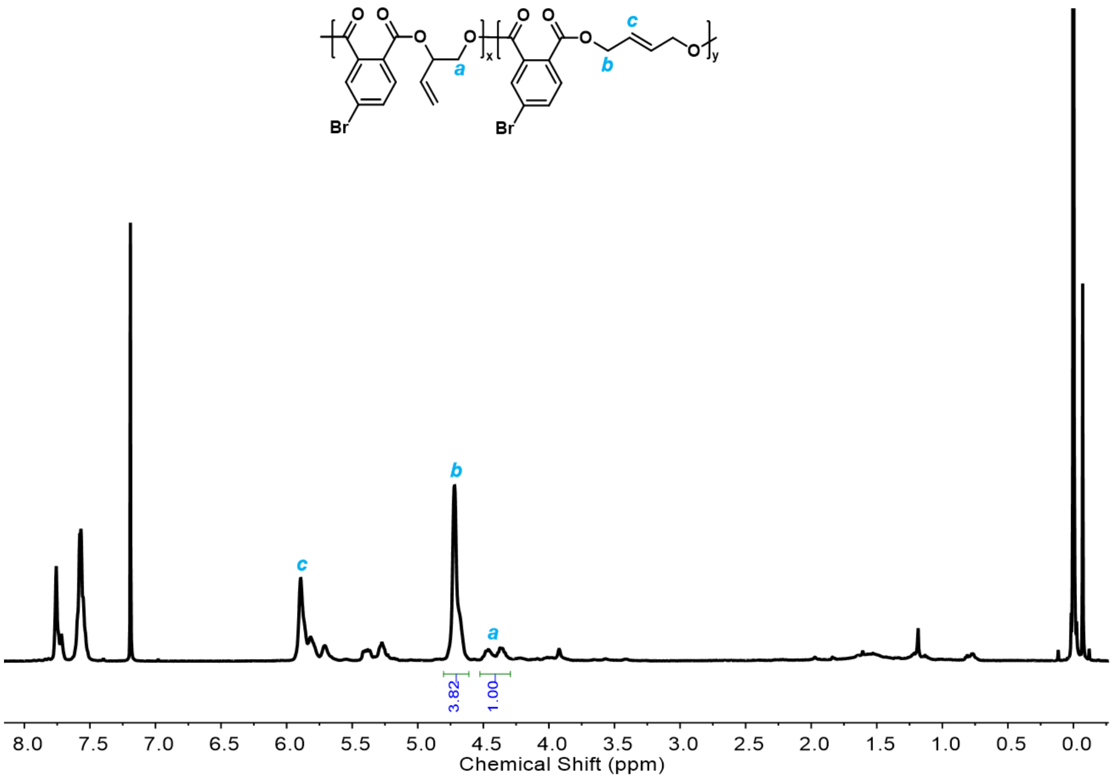

Figure S25. ^{1}H NMR spectrum for polyester **r-P5** ($CDCl_3$). The conversion rate of [3,3]-sigmatropic oxo-rearrangement was 65.6%. The rearrangement conversion = H_b / (H_b + 2H_a) * 100% = 65.6%, H_a and H_b are the integral ratios of peaks a and b, respectively.

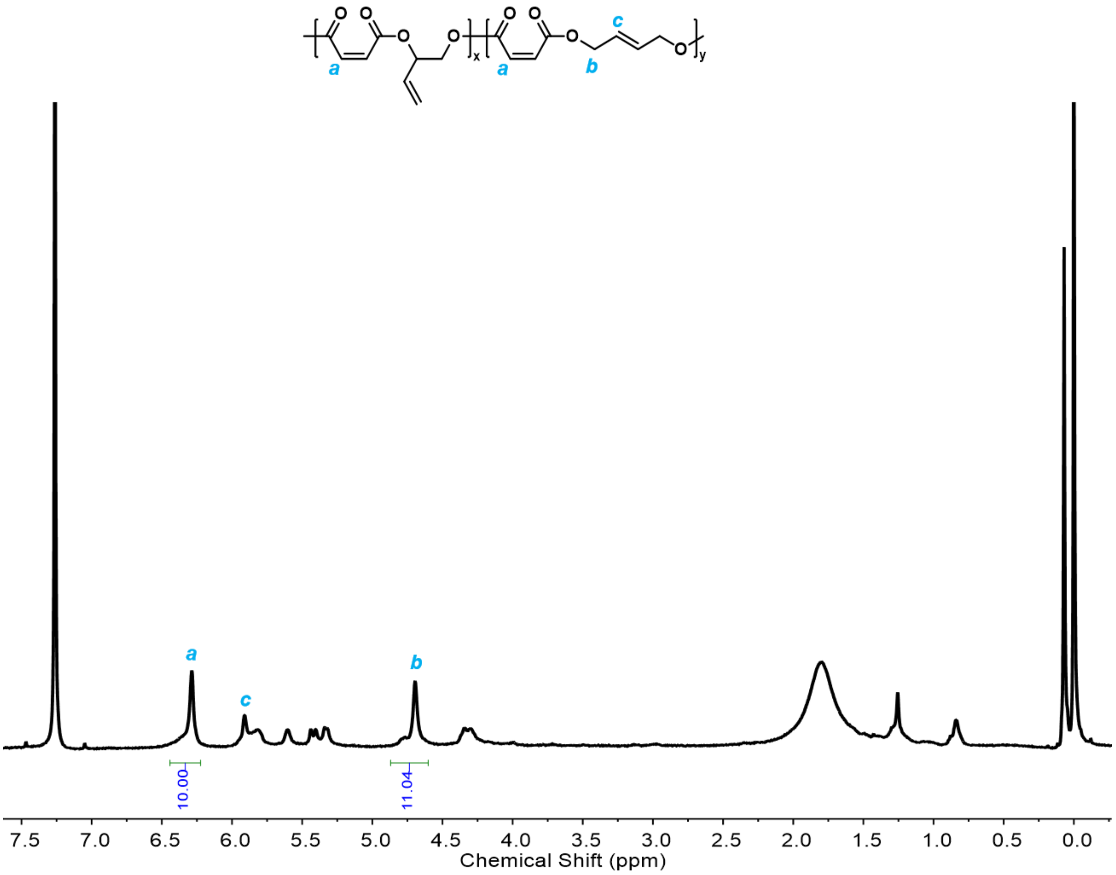

Figure S26. ^{1}H NMR spectrum for polyester **r-P6** ($CDCl_3$). The conversion rate of [3,3]-sigmatropic oxo-rearrangement was 55.2%. The rearrangement conversion = H_b / (H_b + 2H_a) * 100% = 55.2%, H_a and H_b are the integral ratios of peaks a and b, respectively.

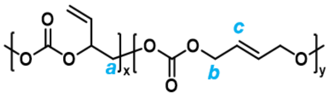

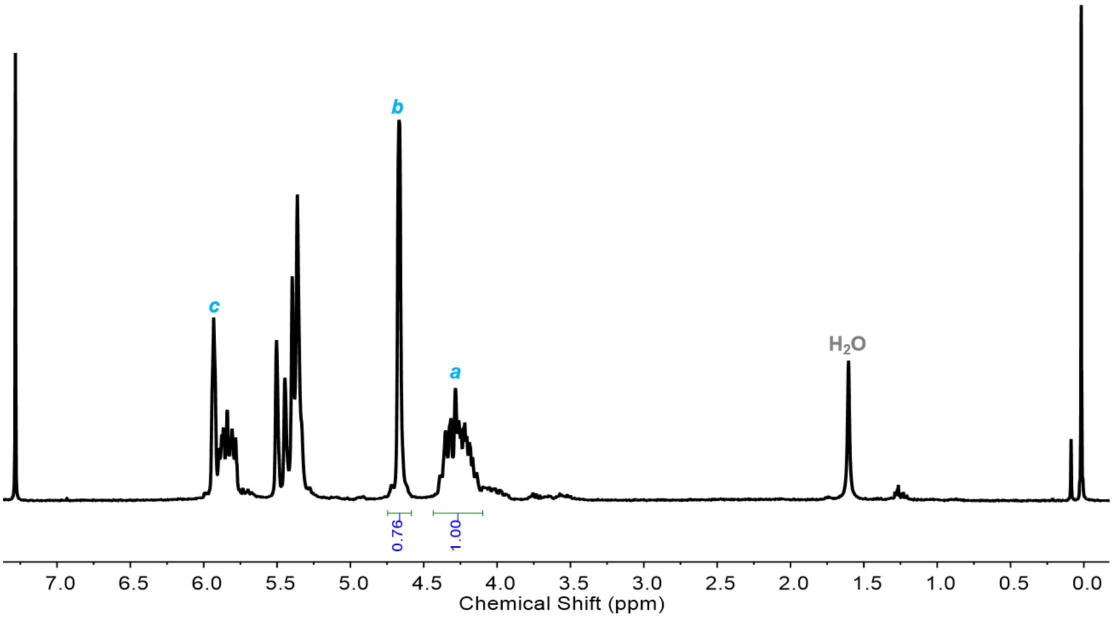

Figure S27. ^{1}H NMR spectrum for polyester **r-P7** ($CDCl_3$). The conversion rate of [3,3]-sigmatropic oxo-rearrangement was 27.5%. The rearrangement conversion = H_b / (H_b + 2H_a) * 100% = 27.5%, H_a and H_b are the integral ratios of peaks a and b, respectively.

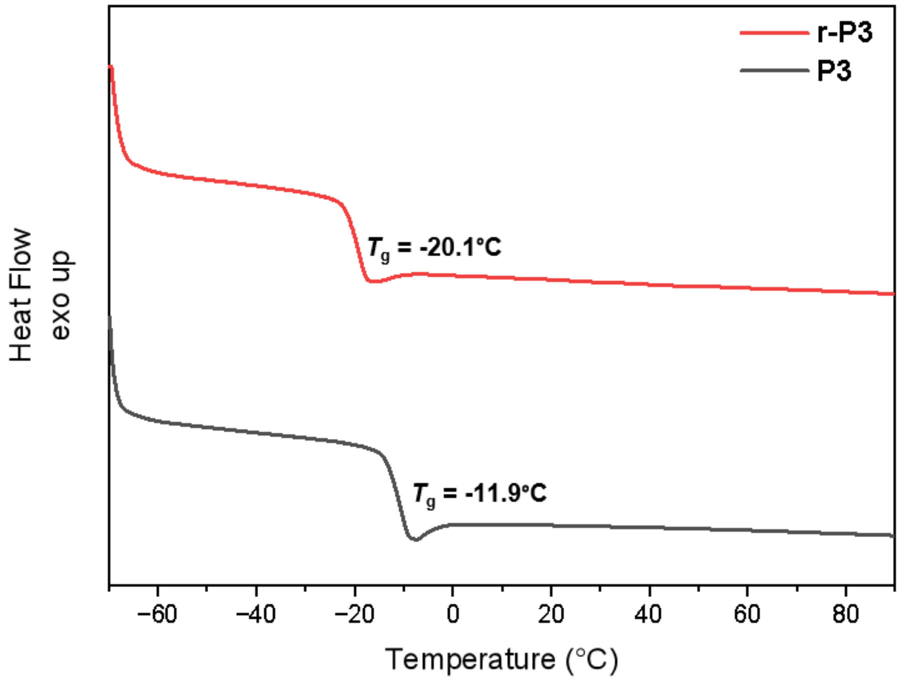

Figure S28. DSC traces (second heating runs) of **P3** and **r-P3**.

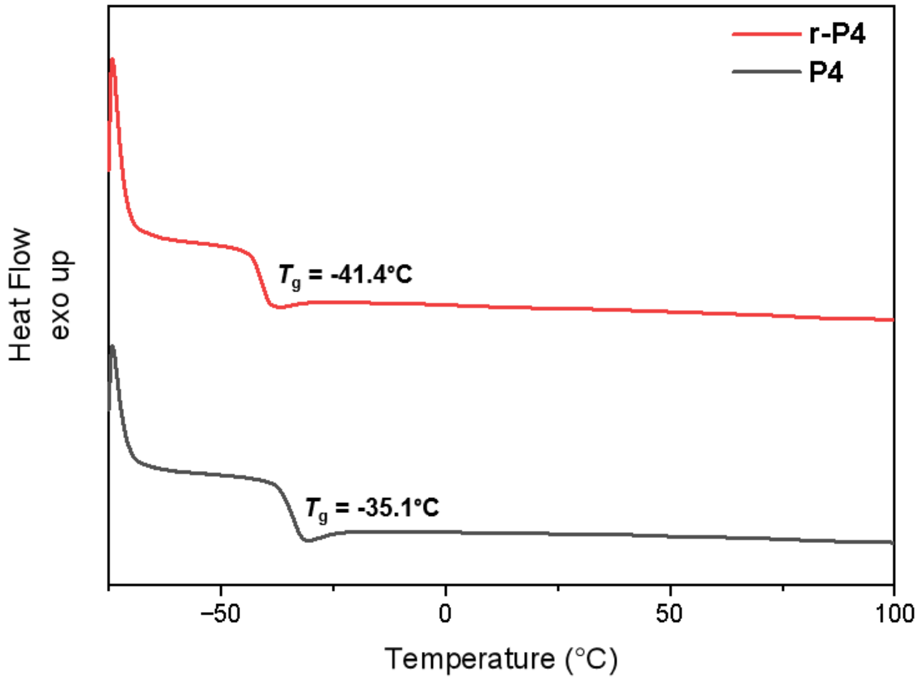

Figure S29. DSC traces (second heating runs) of **P4** and **r-P4**.

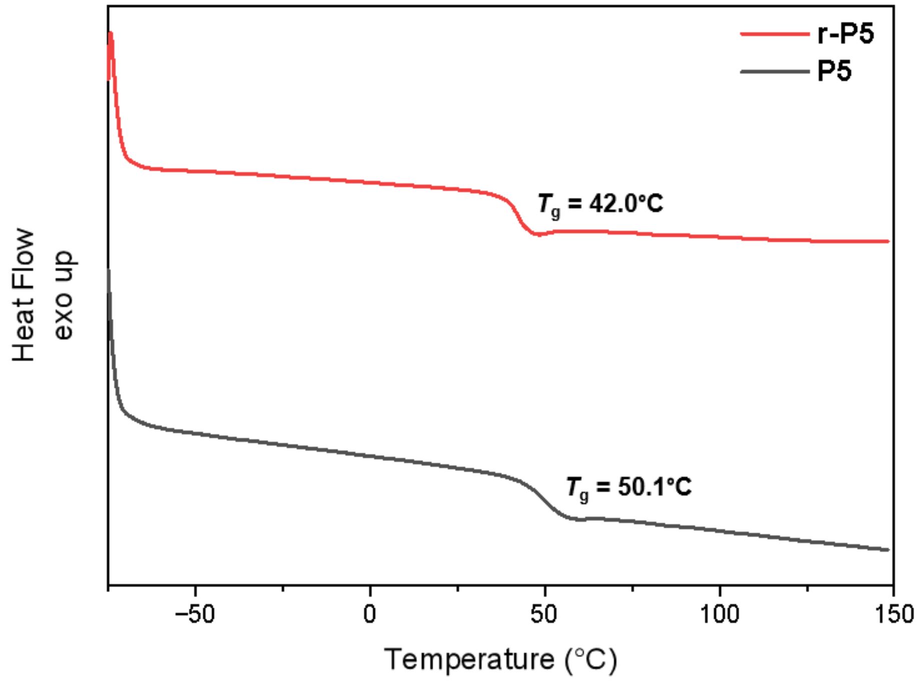

Figure S30. DSC traces (second heating runs) of **P5** and **r-P5**.

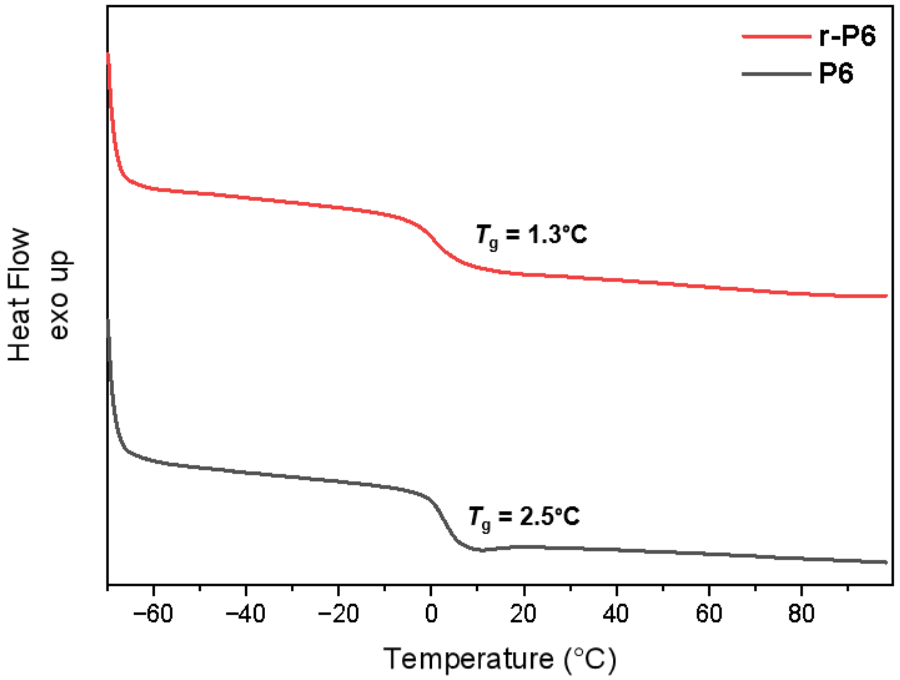

Figure S31. DSC traces (second heating runs) of **P6** and **r-P6**.

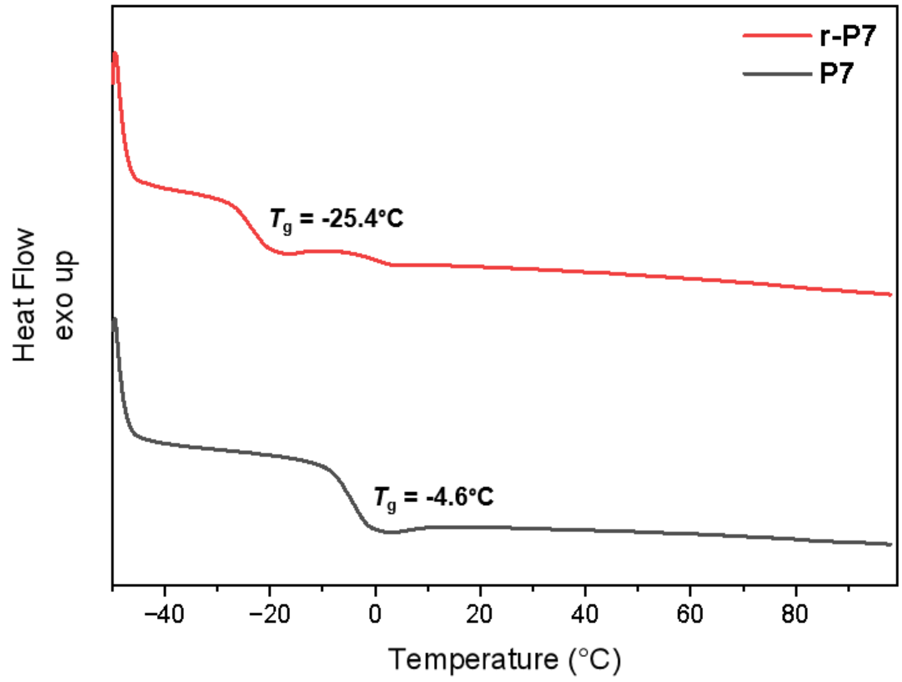

Figure S32. DSC traces (second heating runs) of **P7** and **r-P7**.

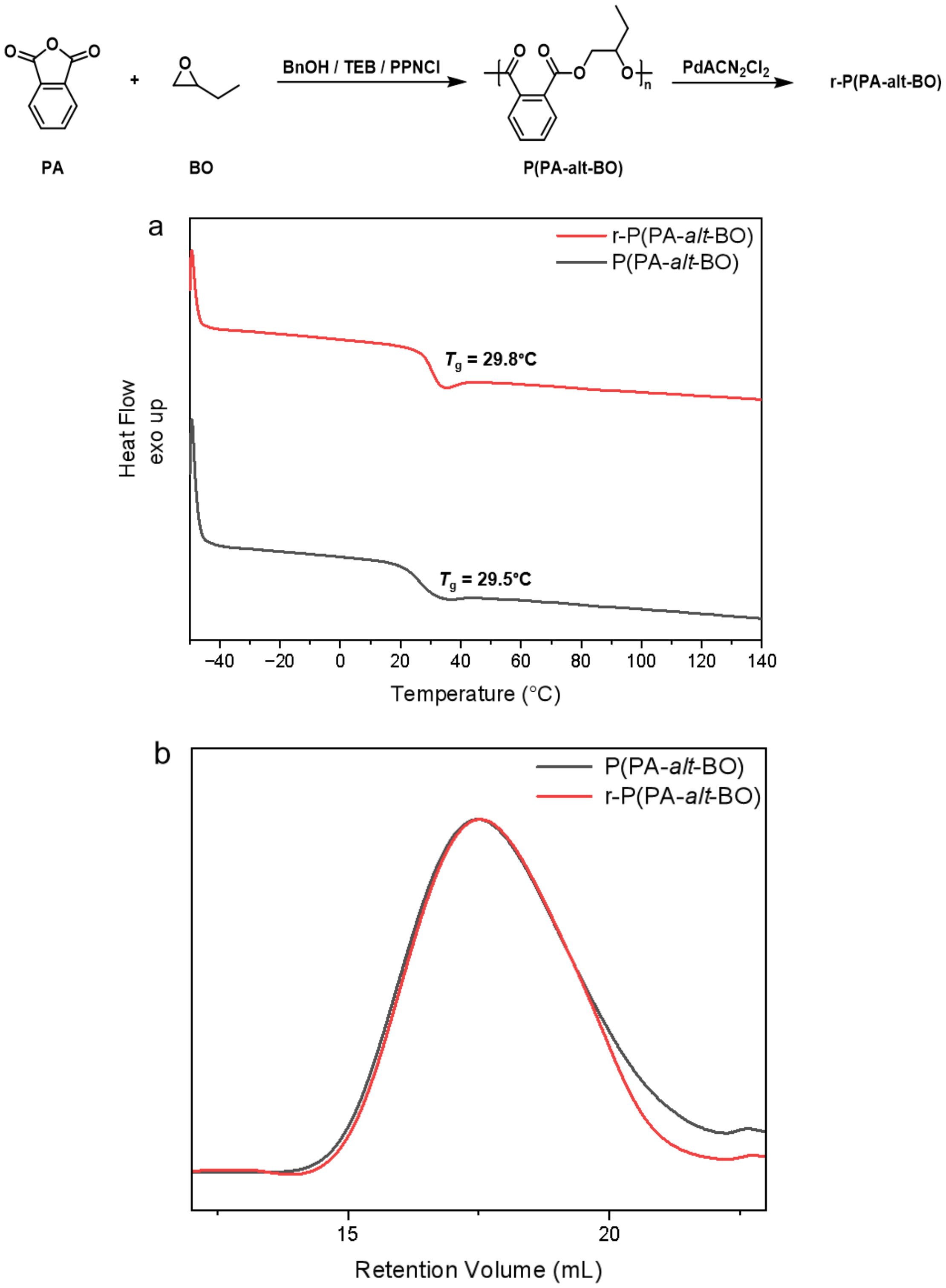

Figure S33. (a) DSC traces (second heating runs) of P(PA-alt-BO) and r-P(PA-alt-BO). (b) Normalized GPC traces of P(PA-alt-BO) and r-P(PA-alt-BO). P(PA-alt-BO) was synthesized by ROCOP of PA and BO with a ratio of PA/BO/TEB/PPNCl/BnOH = 200/300/1.5/1/5 at 80°C for 4 hours. The polymer r-P(PA-alt-BO) was addressed by Pd(CH_3CN_2)Cl_2 at the same condition in **Table 1** entry 1.

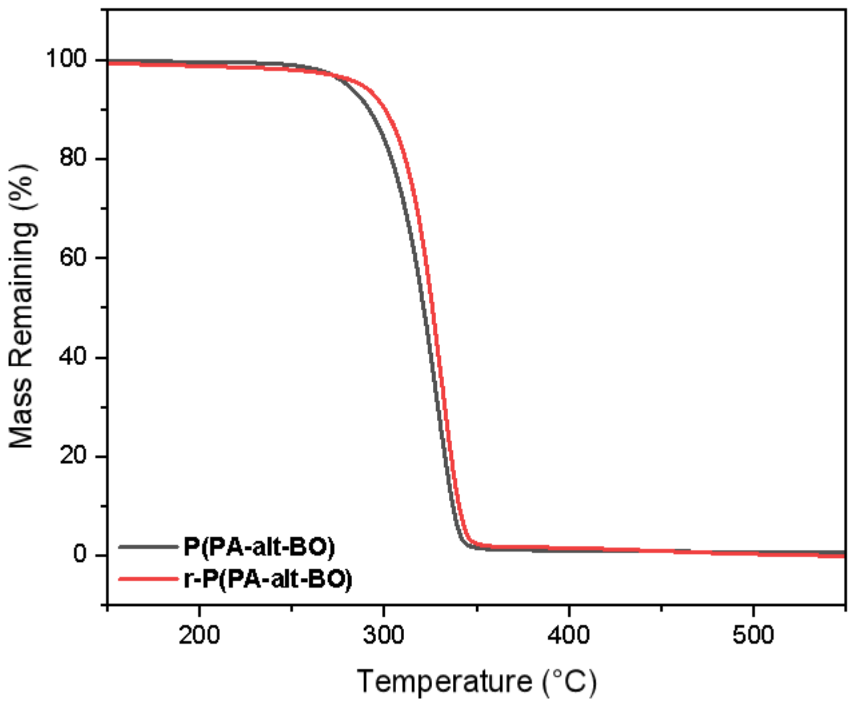

Figure S34. TGA thermograms of polyesters P(PA-alt-BO) and its corresponding rearranged polymers r-P(PA-alt-BO) (Treated with Pd at the condition on **Table 1** entry1).

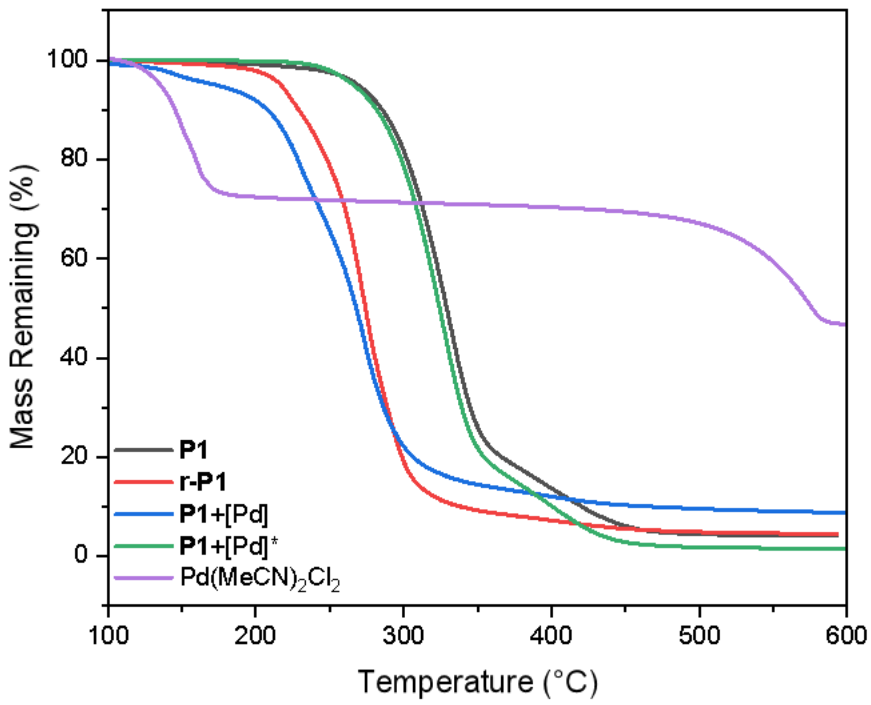

Figure S35. TGA thermograms of polyesters **P1**, **r-P1**, P1+[Pd] (the mixture of 10% [Pd] and **P1**), **P1**+[Pd] (the mixture of 10% [Pd] treated by heating to 300°C and **P1**) and $Pd(CH_3CN)_2Cl_2$.

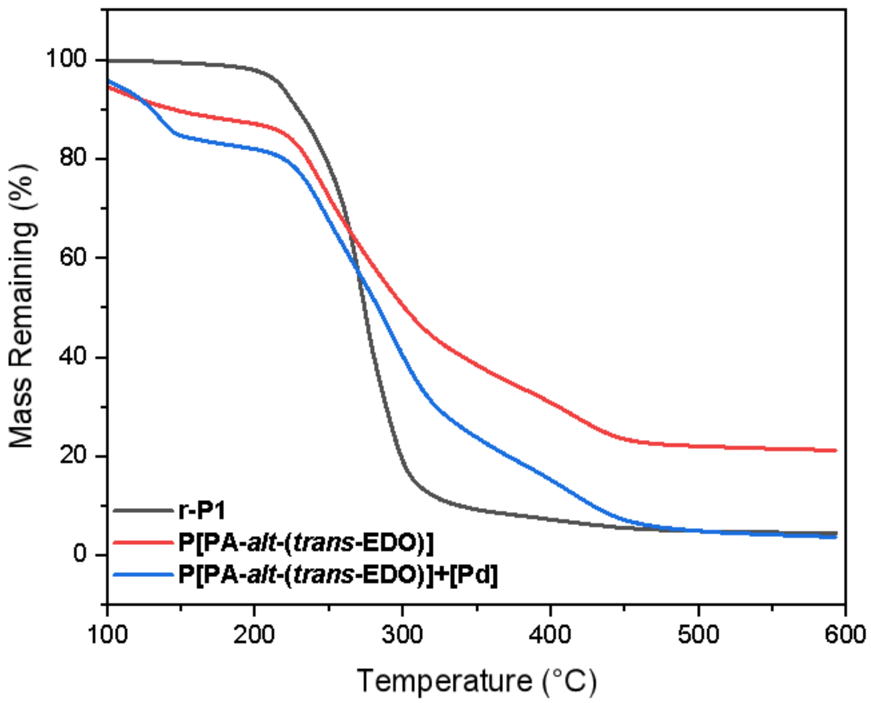

Figure S36. TGA thermograms of polyesters **r-P1**, P[PA-*alt*-(*trans*-BDO)] (prepared from polycondensation of (PA and *trans*-BDO), and P[PA-*alt*-(*trans*-BDO)] + [Pd] (the mixture of 10% [Pd] and P[PA-*alt*-(*trans*-BDO)]).

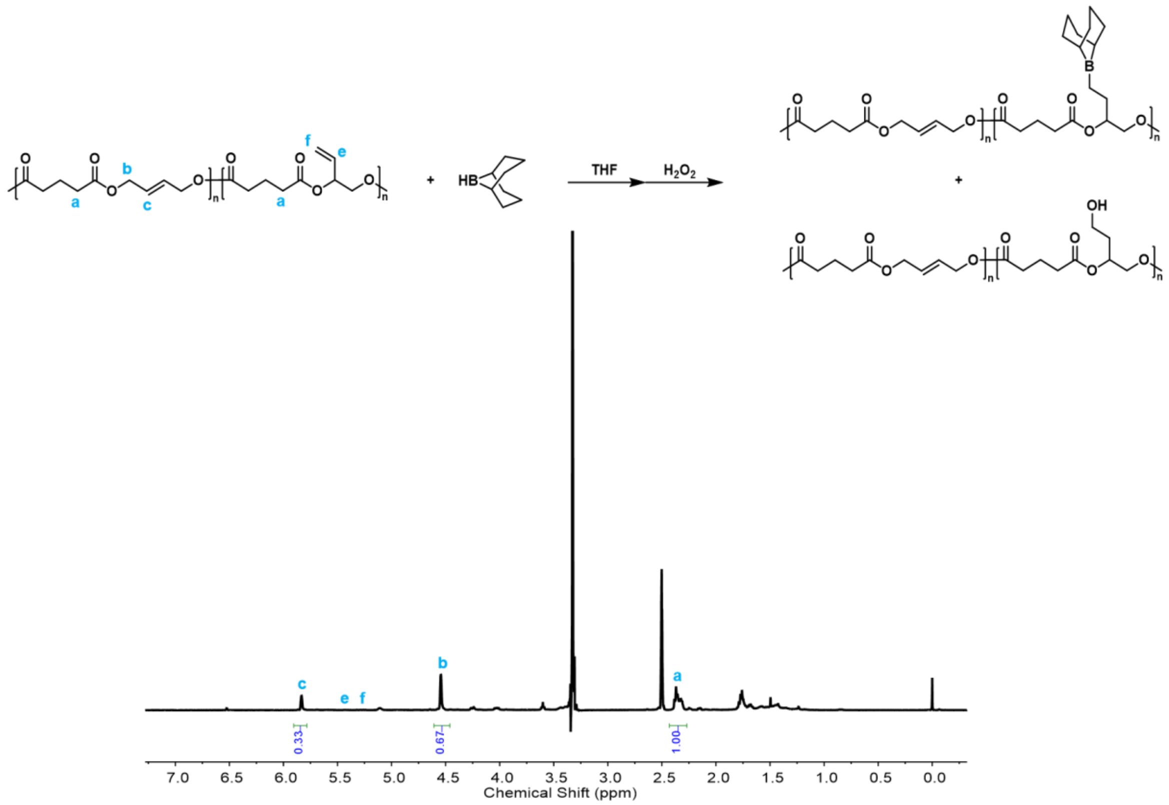

Figure S37. ^{1}H NMR spectrum for the products obtained from **r-P2** addressed by BBN and treated with 30% H_2O_2 aqueous solution. Among them, H_e and H_f were not observed while the integral ratios of peaks a, b and c remained unchanged.

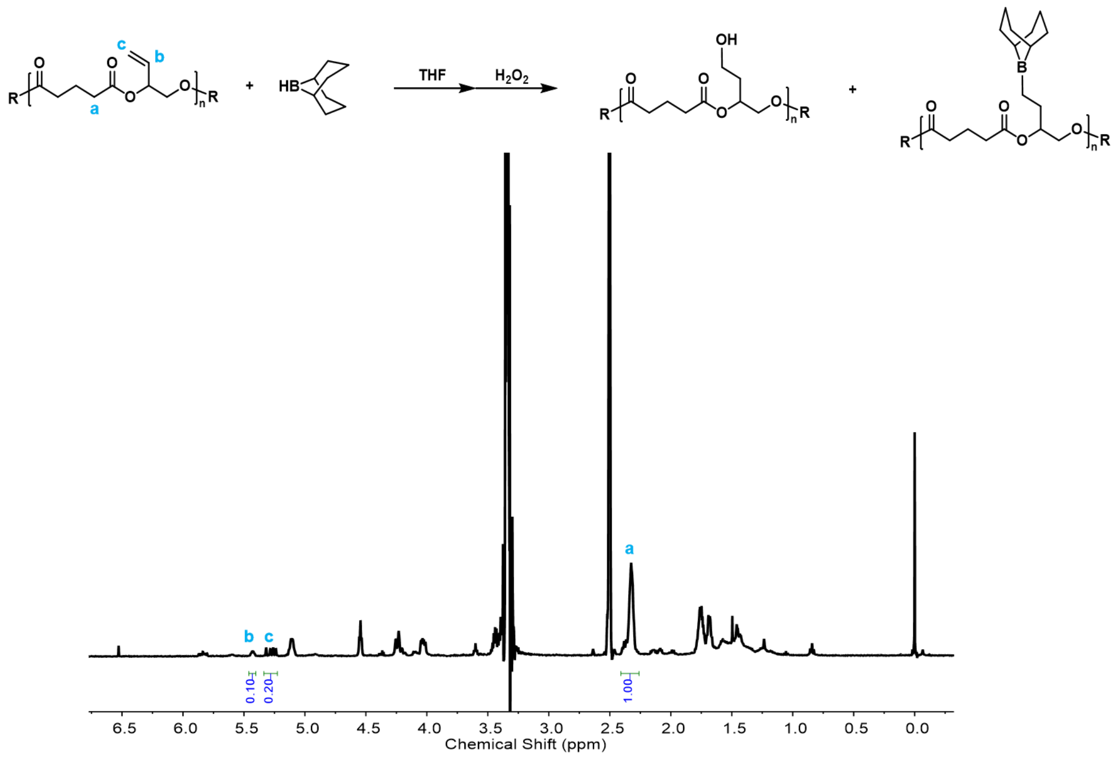

Figure S38. ^{1}H NMR spectrum for the products obtained from **P2** addressed by BBN and treated with 30% H_2O_2 aqueous solution. The conversion rate of terminal alkenes = H_b / H_a * 100% = 80%, H_a and H_b are the integral ratio of the protons a and b peaks, respectively.

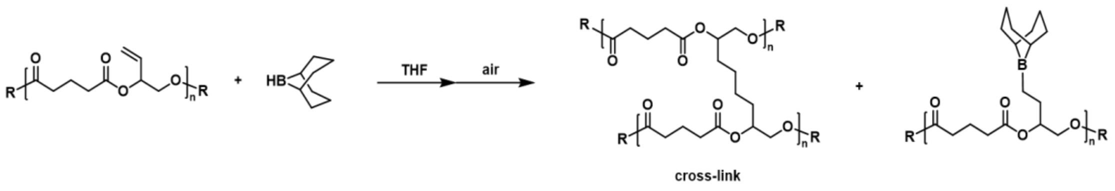

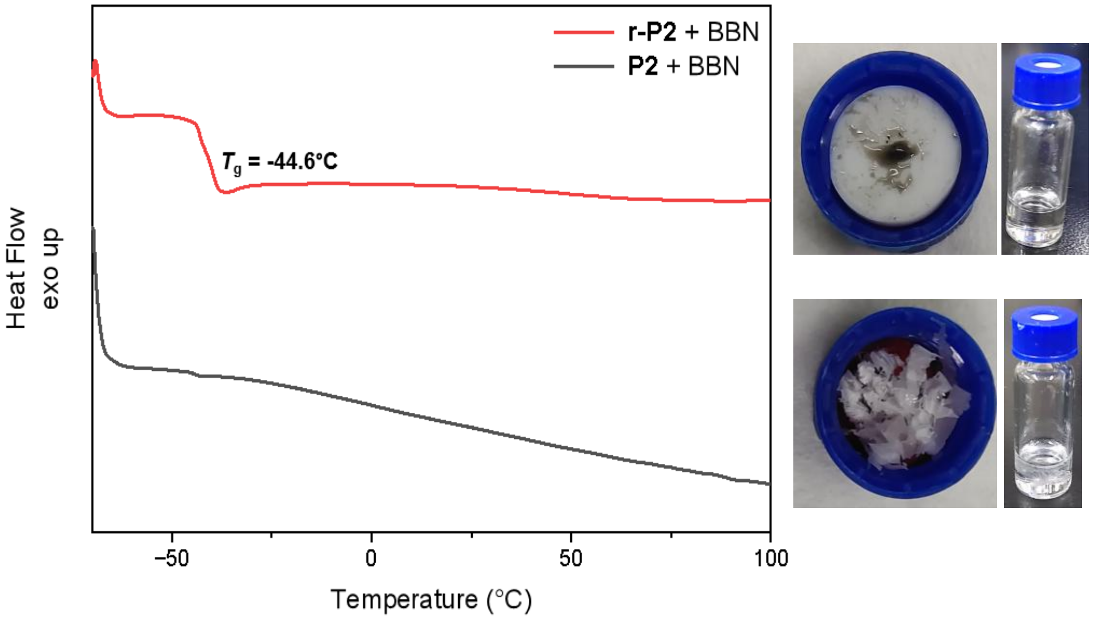

Figure S39. DSC traces (second heating runs) of the products obtained from **P2** or **r-P2** addressed by BBN. The T_g was not observed for the product of **P2** and it exists in a solid state and undergoes swelling in DCM. Whereas **r-P2** can be dissolved in DCM as the photo shows.

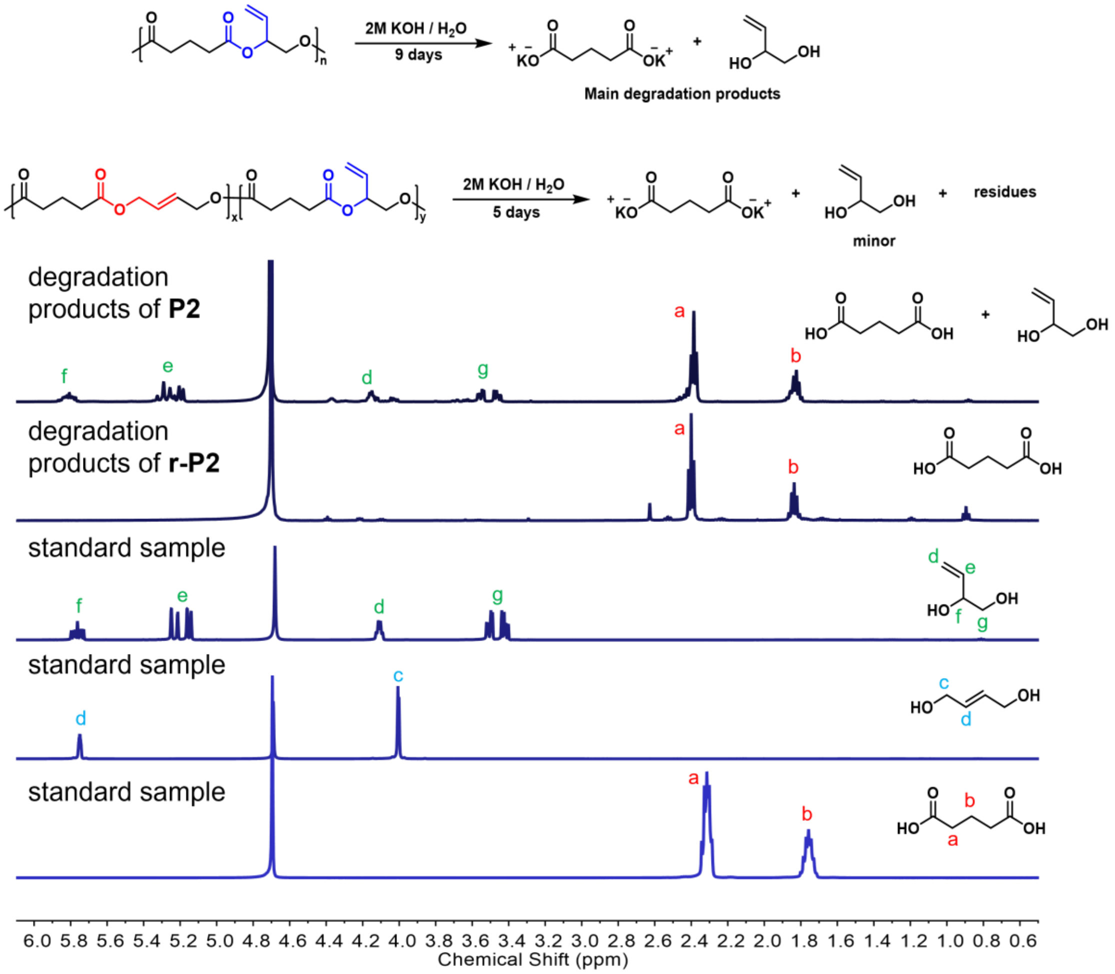

Figure S40. ^{1}H NMR spectrum for the complete degradation products of **P2** and **r-P2** (D_2O).

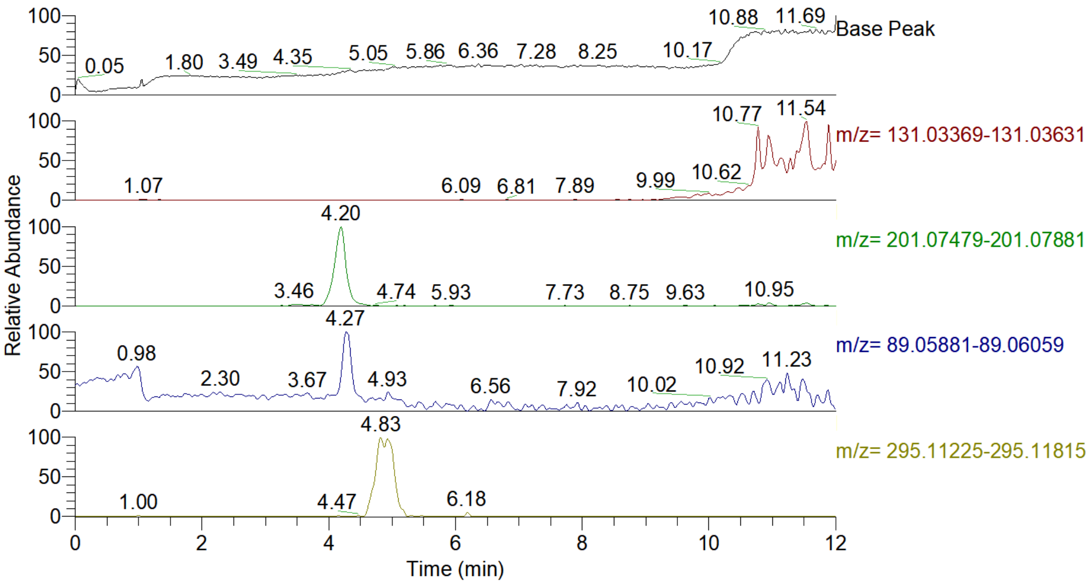

Figure S41. LC-MS Chromatogram of the complete degradation products of **P2**. Degradation conditions: **P2** in KOH (2 M), 60 °C, 9 days.

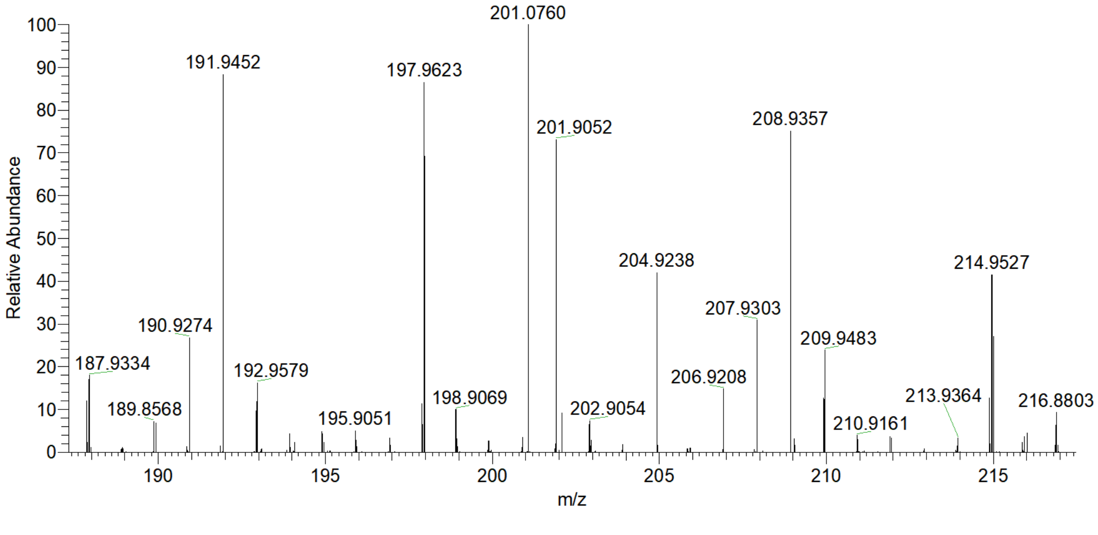

Figure S42. Negative ESI-MS spectrum of the complete degradation products of **P2** degradation at 4.08 - 4.22 minutes.

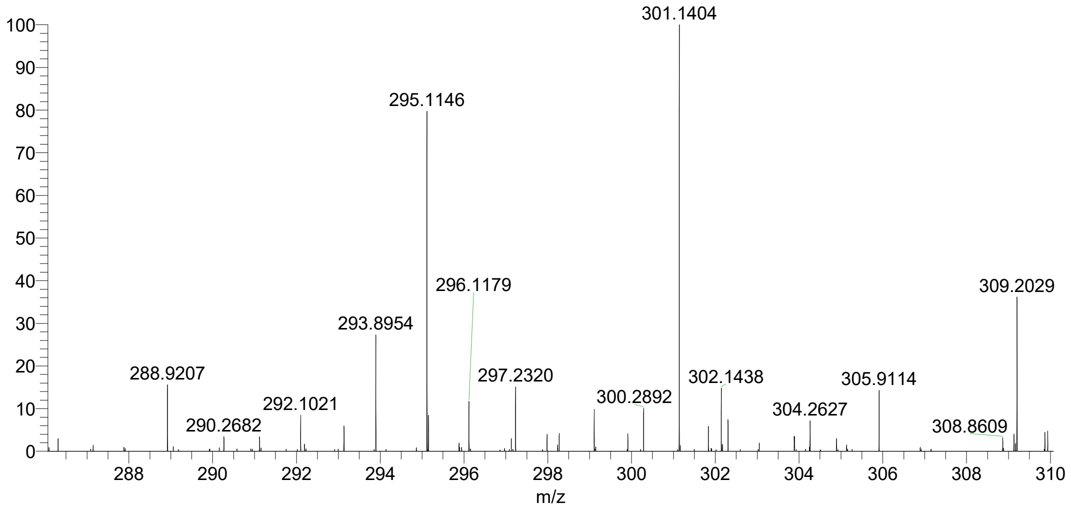

Figure S43. Positive ESI-MS spectrum of the complete degradation products of **P2** degradation at 4.17 - 4.35 minutes.

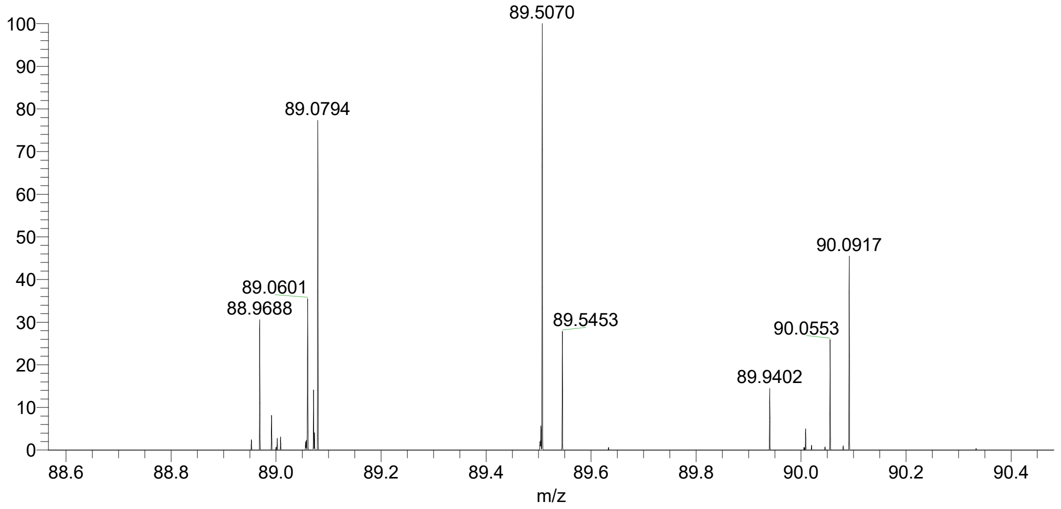

Figure S44. Positive ESI-MS spectrum of the complete degradation products of **P2** degradation at 4.77 - 4.94 minutes.

Table S2. Data and Assignments of the analytes observed from the ESI-MS spectrum for the degradation products of **P2**.

**Product (M)**	**Observed Mass**	**Analyte**	**Expected Mass**	**RT (min)**
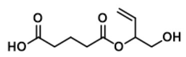	201.0760	$[M-H]^{1-}$	201.0763	4.20
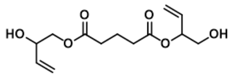	295.1146	$[M-Na]^{1-}$	295.1158	4.83
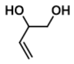	89.0601	$[M+H]^{1+}$	89.0603	4.27

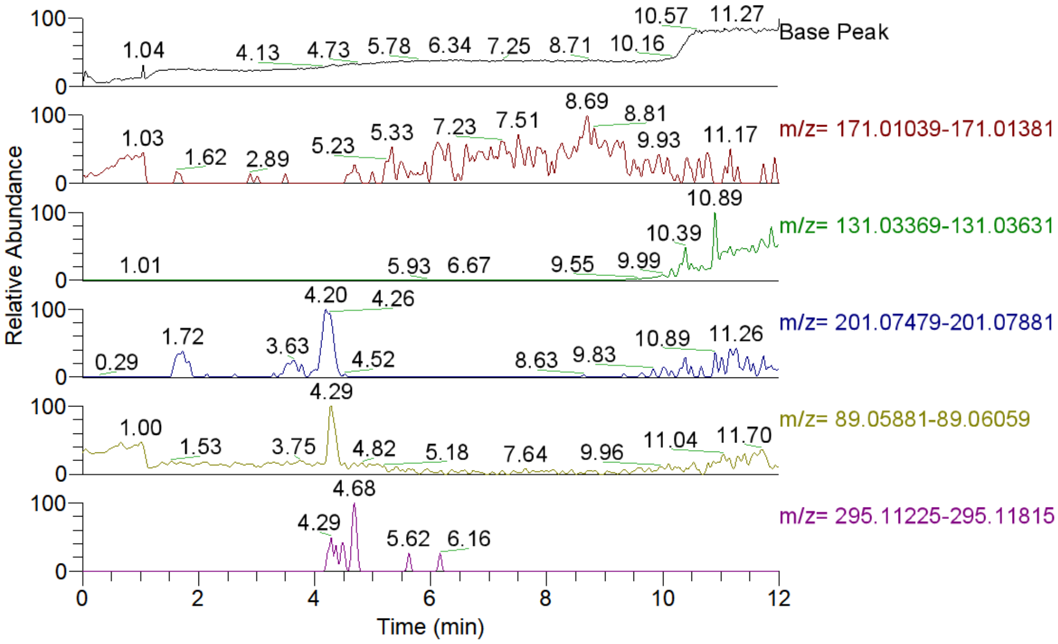

Figure S45. LC-MS Chromatogram of the complete degradation products of **r-P2**. Degradation conditions: **r-P2** in KOH (2 M), 60 °C, 5 days.

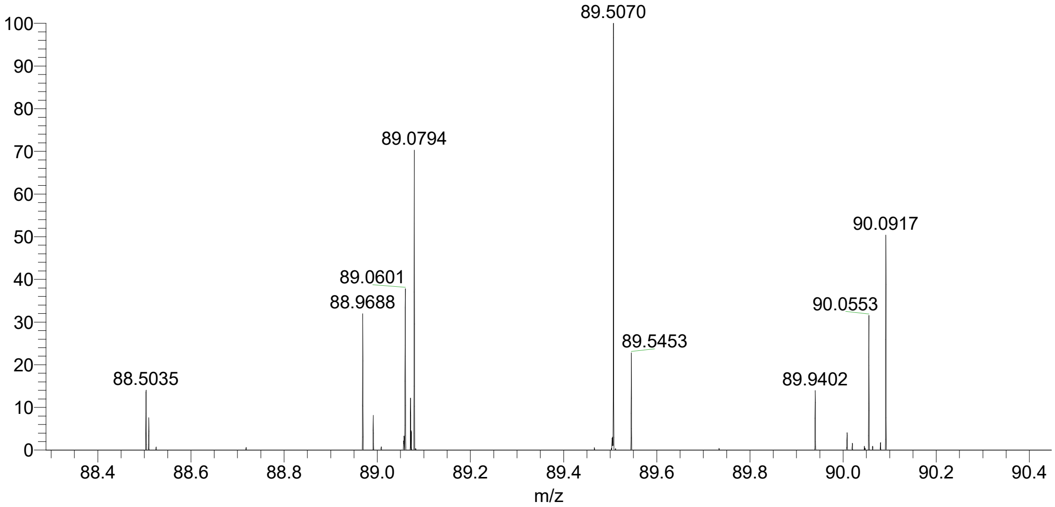

Figure S46. Positive ESI-MS spectrum of the complete degradation products of **r-P2** at 4.21 - 4.35 minutes.

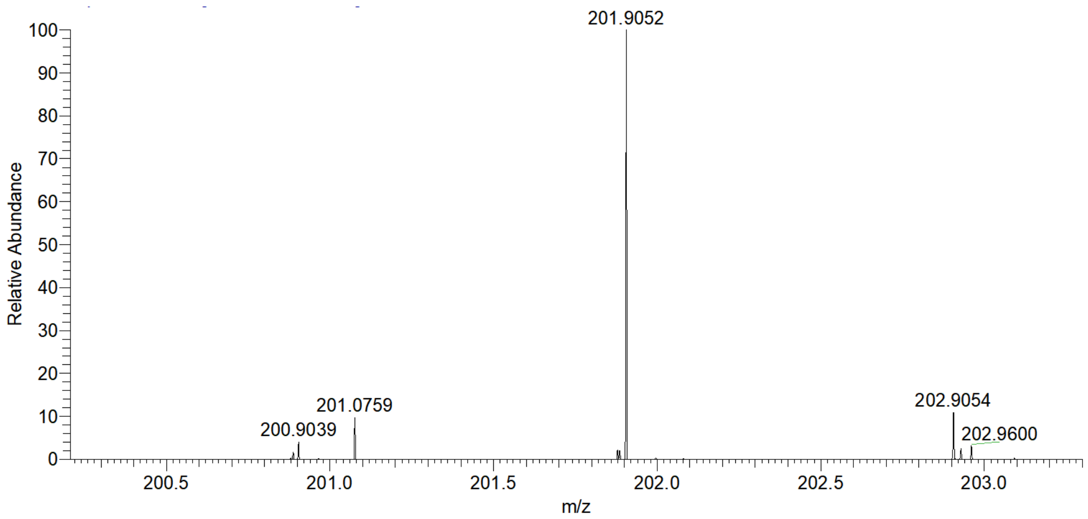

Figure S47. Negative ESI-MS spectrum of the complete degradation products of **r-P2** at 4.21 - 4.35 minutes.

Table S3. Data and Assignments of the analytes observed from LC-MS spectrum for the degradation products of **r-P2**.

**Product (M)**	**Observed Mass**	**Analyte**	**Expected Mass**	**RT (min)**
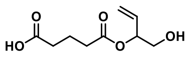	201.0759	$[M-H]^{1-}$	201.0763	4.20
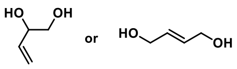	89.0601	$[M+H]^{1+}$	89.0603	4.27

References

1. Longo JM, Sanford MJ, Coates GW. Ring-Opening Copolymerization of Epoxides and Cyclic Anhydrides with Discrete Metal Complexes: Structure-Property Relationships. *Chem Rev*. 2016; **116**(24): 15167-15197.
2. Liang X, Lv J, Qiang H *et al.* Easy access to amphiphilic nitrogenous block copolymers via switchable catalysis. *Chem Sci*. 2024; **15**(44): 18650-18658. doi: 10.1039/D4SC05047A
3. Yi N, Chen TTD, Unruangsri J *et al.* Orthogonal functionalization of alternating polyesters: selective patterning of (AB)n sequences. *Chem Sci*. 2019; **10**(43): 9974-9980. doi: 10.1039/c9sc03756j
4. Chen K, Shi G, Li H *et al.* Design of Betaine Functional Catalyst for Efficient Copolymerization of Oxirane and CO2. *Macromolecules*. 2018; **51**(15): 6057-6062. doi: 10.1021/acs.macromol.8b01103
5. Zhang C, Geng X, Zhang X *et al.* Alkyl borane-mediated metal-free ring-opening (co)polymerizations of oxygenated monomers. *Prog Polym Sci*. 2023; **136**: 101644.
6. Braddock DC, Matsuno A. In situ tandem allylic acetate isomerisation-ring closing metathesis: 1,3-dimesityl-4,5-dihydroimidazol-2-ylidene ruthenium benzylidenes and palladium(0)–phosphine combinations. *Tetrahedron Lett*. 2002; **43**(18): 3305-3308.
7. Nomura N, Tsurugi K, Okada M. Palladium-Catalyzed Allylic Substitution Polymerization: Polycondensation of Carbon−Carbon Bond Formation by Linkage between Csp3 and Csp3 of Monomers. *J Am Chem Soc*. 1999; **121**(31): 7268-7269. doi: 10.1021/ja991211a
8. Oehlschlager C, Mishra P, Dhami S. Metal-catalyzed rearrangements of allylic esters. *Can J Chem*. 1984; **62**(4): 791-797. doi: 10.1139/v84-132
9. Ditzler RAJ, Rapagnani RM, Berney NK *et al.* Architectural Editing of Polyesters and Polyurethanes via Palladium(II)-Catalyzed [3,3]-Sigmatropic Oxo-Rearrangements. *J Am Chem Soc*. 2024; **146**(22): 15286-15292. doi: 10.1021/jacs.4c02917
10. Martín Castro AM. Claisen Rearrangement over the Past Nine Decades. *Chem Rev*. 2004; **104**(6): 2939-3002. doi: 10.1021/cr020703u
11. Lv C, Du Y, Pan X. Alkylboranes in Conventional and Controlled Radical Polymerization. *J Polym Sci*. 2020; **58**(1): 14-19. doi: https://doi.org/10.1002/pola.29477
12. Cai Q, Bai T, Zhang H *et al.* Catalyst-free synthesis of polyesters via conventional melt polycondensation. *Materials Today*. 2021; **51**: 155-164. doi: 10.1016/j.mattod.2021.07.024
13. Deacy AC, Gregory GL, Sulley GS *et al.* Sequence Control from Mixtures: Switchable Polymerization Catalysis and Future Materials Applications. *J Am Chem Soc*. 2021; **143**(27): 10021-10040. doi: 10.1021/jacs.1c03250
14. Ratushnyy M, Zhukhovitskiy AV. Polymer Skeletal Editing via Anionic Brook Rearrangements. *J Am Chem Soc*. 2021; **143**(43): 17931-17936. doi: 10.1021/jacs.1c06860
15. Rapagnani RM, Dunscomb RJ, Fresh AA, Tonks IA. Tunable and recyclable polyesters from CO(2) and butadiene. *Nat Chem*. 2022; **14**(8): 877-883. doi: 10.1038/s41557-022-00969-2
